# Hexagonal Plasmonic Arrays for High-Throughput Multicolor Single-Molecule Studies

**DOI:** 10.1021/acsami.4c04744

**Published:** 2024-07-23

**Authors:** Ediz Kaan Herkert, Lukas Lau, Roger Pons Lanau, Maria F. Garcia-Parajo

**Affiliations:** † ICFO - Institut de Ciencies Fotoniques, The Barcelona Institute of Science and Technology, 08860 Castelldefels, Barcelona, Spain; ‡ ICREA, 08010 Barcelona, Spain

**Keywords:** nanophotonic biosensors, single-molecule detection, multicolor fluorescence detection, high-throughput sensing, plasmonics

## Abstract

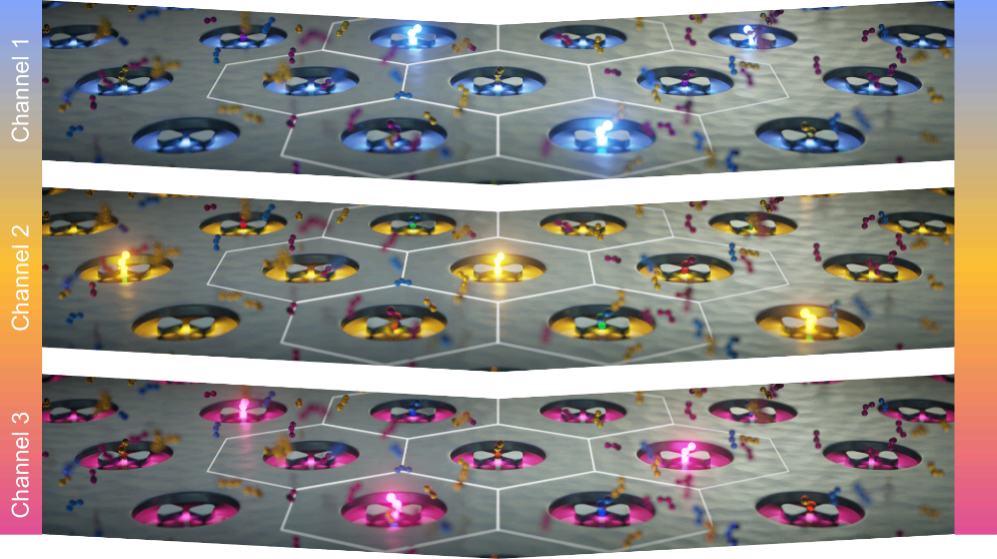

Nanophotonic biosensors offer exceptional sensitivity in the presence of strong background signals by enhancing and confining light in subwavelength volumes. In the field of nanophotonic biosensors, antenna-in-box (AiB) designs consisting of a nanoantenna within a nanoaperture have demonstrated remarkable single-molecule fluorescence detection sensitivities under physiologically relevant conditions. However, their full potential has not yet been exploited as current designs prohibit insightful correlative multicolor single-molecule studies and are limited in terms of throughput. Here, we overcome these constraints by introducing aluminum-based hexagonal close-packed AiB (HCP-AiB) arrays. Our approach enables the parallel readout of over 1000 HCP-AiBs with multicolor single-molecule sensitivity up to micromolar concentrations using an alternating three-color excitation scheme and epi-fluorescence detection. Notably, the high-density HCP-AiB arrays not only enable high-throughput studies at micromolar concentrations but also offer high single-molecule detection probabilities in the nanomolar range. We demonstrate that robust and alignment-free correlative multicolor studies are possible using optical fiducial markers even when imaging in the low millisecond range. These advancements pave the way for the use of HCP-AiB arrays as biosensor architectures for high-throughput multicolor studies on single-molecule dynamics.

## 1 Introduction

The ability to optically detect single molecules has enabled the studies of biochemical dynamics beyond of what can be learned from ensemble averages.^1,2^ Capturing the heterogeneity of biological systems at the single-molecule level is crucial to reveal the function of various biomolecules within their complex biological environment. The use of fluorescent labels is nowadays the most common approach to detecting individual biomolecules.^3,4^ Advanced single-molecule detection techniques like single-particle tracking (SPT) employ such fluorescent labels to unveil the dynamics of distinct biomolecules in living cells.^5^ However, monitoring dynamic interactions between biomolecules often requires micro- to millimolar concentrations to guarantee that these interactions occur frequently enough to be observed. Unfortunately, diffraction-limited techniques cannot isolate single molecules at these high concentrations.^6^ Because of this, there is a need for techniques that offer single-molecule detection sensitivity in this concentration range by providing subdiffraction observation volumes. Single molecule-based super-resolution methods circumvent the diffraction limit, but their poor temporal resolution prevents the visualization of single molecule dynamics in real time. Stimulated emission depletion (STED) microscopy provides subdiffraction excitation volumes and can be implemented to provide dynamic information on biomolecules at much higher concentrations as compared to diffraction-limited confocal illumination.^7^ However, STED is often unsuitable for single-molecule studies as it requires the use of high laser powers which lead to increased fluorophore photobleaching and could compromise cell viability.

In 2003, Levene et al. demonstrated that fluorescence correlation spectroscopy (FCS) with aluminum zero-mode waveguides (ZMWs) can enable single-molecule studies at micromolar concentrations.^8^ More recently, ZMWs were used to perform dual-color cross-correlation studies with living cells.^9^ ZMWs were undoubtedly the key to many more important single-molecule studies with the real-time sequencing of DNA being arguably the most impactful one.^10−12^ However, the strongly reduced observation volumes enabling these single-molecule studies at micromolar concentrations come at the cost of severely reduced fluorescence signals due to the weak fluorescence excitation provided by ZMWs and the fluorescence quenching that fluorophores can experience near metals.^13,14^ The fluorescence quenching can reduce the fluorescence emission to an extent that single-molecule sensitivity is not ensured anymore.

In contrast, plasmonic nanoantennas offer both subdiffraction observation volumes and strongly enhanced fluorescence emission rates for a fluorophore within the nanoantenna hotspot region.^15^ The enhanced fluorescence emission rate comes down to two key factors. First, the excitation rate of the fluorophore is increased due to the enhanced excitation intensity in the nanoantenna hotspot. Second, the decay rates of the fluorophore in the hotspot are increased through the Purcell effect.^14,16,17^ The latter can increase the quantum efficiency especially for fluorophores with a low intrinsic quantum efficiency. However, the diffraction-limited excitation light necessary to excite the nanoantenna plasmon resonance generates a significant fluorescence background that often conceals the enhanced single-molecule signal from the nanoantenna hotspot. To sufficiently mitigate this effect at high fluorophore concentrations, studies based on nanoantennas typically require specimens with axial extents of only a few nanometers, (chemically quenched) fluorophores with very low quantum yields, or both.^15,18^ Unfortunately, these requirements cannot always be ensured in biosensing applications rendering the use of traditional nanoantennas futile in these cases.

To overcome these challenges, Punj et al. introduced antenna-in-box (AiB) platforms consisting of a nanoantenna within a nanoaperture and thus unifying strong fluorescence background reduction and fluorescence emission enhancement.^19^ Throughout the years, AiB designs have been further refined toward higher single-molecule detection sensitivity and biocompatibility and adapted by various authors for biosensing applications with model and living cell membranes.^20−25^ Despite providing single-molecule sensitivity at concentrations above 20 μM, the wider adaptation of AiBs for biosensing applications is currently hampered by two key reasons. First, current AiB designs are limited to single-color fluorescence applications in the red and near-infrared wavelength range due to the use of gold as plasmonic material.^26^ This not only restricts the range of available fluorophores but most importantly prohibits multicolor cross-correlation studies that investigate the interactions between different biomolecule species. Second, the throughput of biosensing studies with AiBs is severely slowed down by their sequential point-by-point readout making the acquisition of robust statistics tedious. Winkler et al. outlined in an initial study the potential of a parallelized single-color readout of AiB arrays but the sensing throughput is still limited by the low packing density of these arrays.^24^

**Figure 1:**
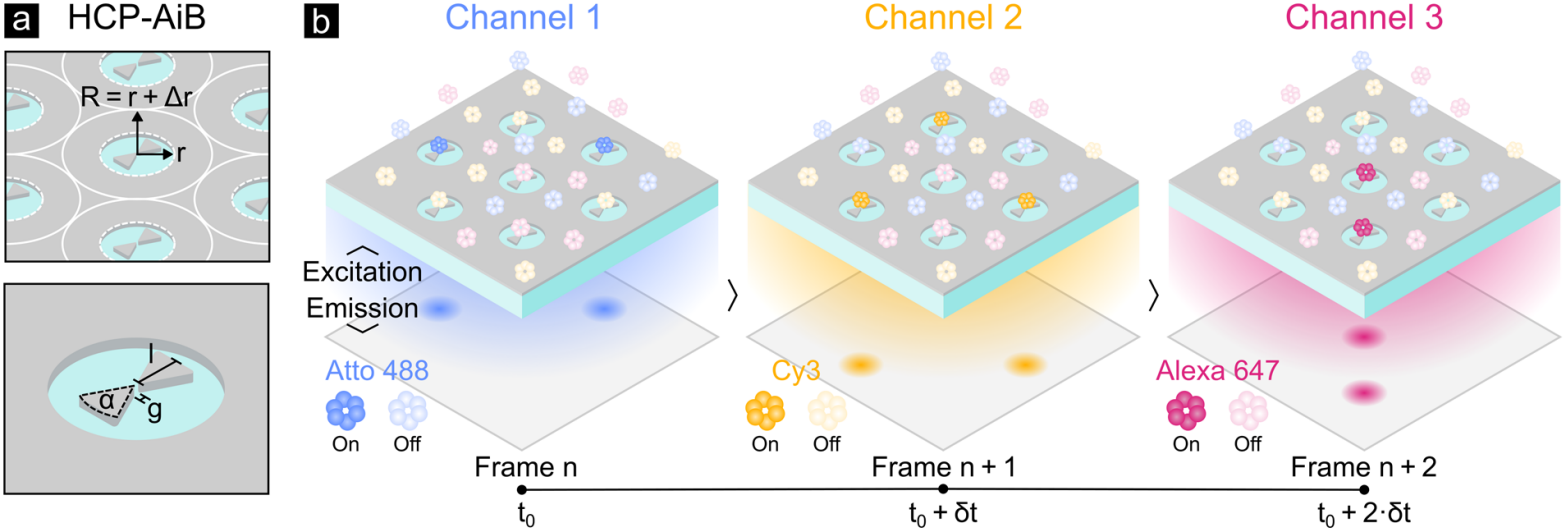
(a) The HCP-AiBs presented here consist of an aluminum bowtie nanoantenna (BNA) within an aluminum nanohole (NH). The BNAs are characterized by their length *l*, gap size *g*, and apex angle α and the NHs by their radius *r*. The HCP arrangement is described by the half center-to-center distance R = *r*+Δ*r* and half edge-to-edge distance Δ*r*. The aluminum film has a height *h*. (b) Here, the capability of HCP-AiBs to provide high single-molecule sensitivity throughout the visible spectrum is exploited through an alternating three-color widefield excitation scheme. The three excitation channels capture the fluorescence emission of the fluorophores Atto 488, Cy3, and Alexa 647, respectively, and are recorded sequentially with a time shift corresponding to the camera exposure time *δt*. This enables high-throughput multicolor single-molecule studies at micromolar concentrations with over 1000 HCP-AiBs in parallel.

Here, we show that high-density hexagonal close-packed AiB (HCP-AiB) arrays made of aluminum provide high-throughput and multicolor single-molecule sensitivity at micromolar concentrations. We discuss the careful computational optimization of the HCP-AiB design and the use of a tailored three-step electron beam lithography (EBL) overlay process to fabricate high-density HCP-AiB arrays. We show that these high-density HCP-AiB arrays enable high-throughput readout by combining them with an alternating three-color widefield excitation scheme and camera-based epi-detection, as sketched in Figure 1. We introduce a robust image analysis pipeline to automatically extract single-molecule traces and to enable alignment-free cross-correlation studies using optical fiducial markers (OFMs). Overall, we demonstrate that the automatic readout of over 1000 HCP-AiBs in parallel ensures high single-molecule detection probabilities at nano- and micromolar concentrations and exposure times as short as 10 ms.

## 2 Results and Discussion

The main goal of this study is to demonstrate the suitability of the HCP-AiB design for high-throughput multicolor single-molecule studies at micromolar concentrations. To achieve this performance, the design of current AiBs must be updated in two major aspects. First, the gold nanoantennas of previous AiB designs must be replaced by nanoantennas of a different material that supports broadband plasmonic resonances throughout the visible range. Second, AiB arrays with significantly higher packing-densities are required to provide an increased number of AiBs on a small readout area, which is important to enable high-speed imaging.

Based on these considerations, we opted for HCP-AiBs consisting of aluminum bowtie nanoantennas (BNAs) within aluminum nanoholes (NHs). Aluminum is our material of choice as it provides broadband plasmonic resonances throughout the visible wavelength range and naturally forms a thin protective oxide layer of about 3 nm.^27−29^ Furthermore, we showed in a recent study that AiBs with an aluminum nanoaperture provide superior fluorescence emission rate enhancement and signal-to-background ratios (SBRs).^25^ Given the round AiB shape, an HCP arrangement is chosen to ensure an optimal packing density.

Figure 1 (a) shows the geometrical parameters required to fully describe the design of HCP-AiBs. The BNA is parametrized by its length *l*, gap size *g*, and apex angle α, the NH by its radius *r*, and the HCP arrangement by the half center-to-center distance R = *r* + *Δr*. The edge-to-edge distance 2·*Δr* is crucial for defining a packing-density limit below which individual AiBs cannot be optically resolved anymore
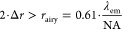
1This packing-density limit is derived from the diffraction limit using the radius *r*_airy_ of the Airy disk at a given fluorescence emission wavelength λ_em_ and numerical aperture NA. Because of this, we choose *Δr* = 250 nm to maintain a small spatial margin also in the red wavelength range using a NA = 1.2 objective. Based on considerations outlined in Section 1 of the Supporting Information, we selected a BNA length of *l* = 80 nm, a gap size of *g* = 20 nm, an apex angle of α = 90°, and an aluminum film of height *h* = 50 nm.

The optimal radius *r* is determined in a more elaborate computational optimization as it has a significant influence on both the fluorescence emission rate and SBR.^25^ Moreover, it is the only parameter that influences the center-to-center distance 2·*R* – given that *Δr* is fixed by the diffraction limit–and therefore affects lattice effects originating from the periodic hexagonal packing. Our optimization objective is to identify the HCP-AiB radius that maximizes the fluorescence emission and minimizes the fluorescence background for the three widely used fluorophores Atto 488 (λ_abs_ = 499 nm, λ_em_ = 520 nm, η_fl_^(0)^ = 0.8), Cy3 (λ_abs_ = 552 nm, λ_em_ = 568 nm, η_fl_^(0)^ = 0.31), and Alexa 647 (λ_abs_ = 653 nm, λ_em_ = 668 nm, η_fl_^(0)^ = 0.15). Here, λ_abs_ is the maximal absorption wavelength, λ_em_ the maximal emission wavelength, and η_fl_^(0)^ the intrinsic quantum efficiency of the fluorophore.

**Figure 2:**
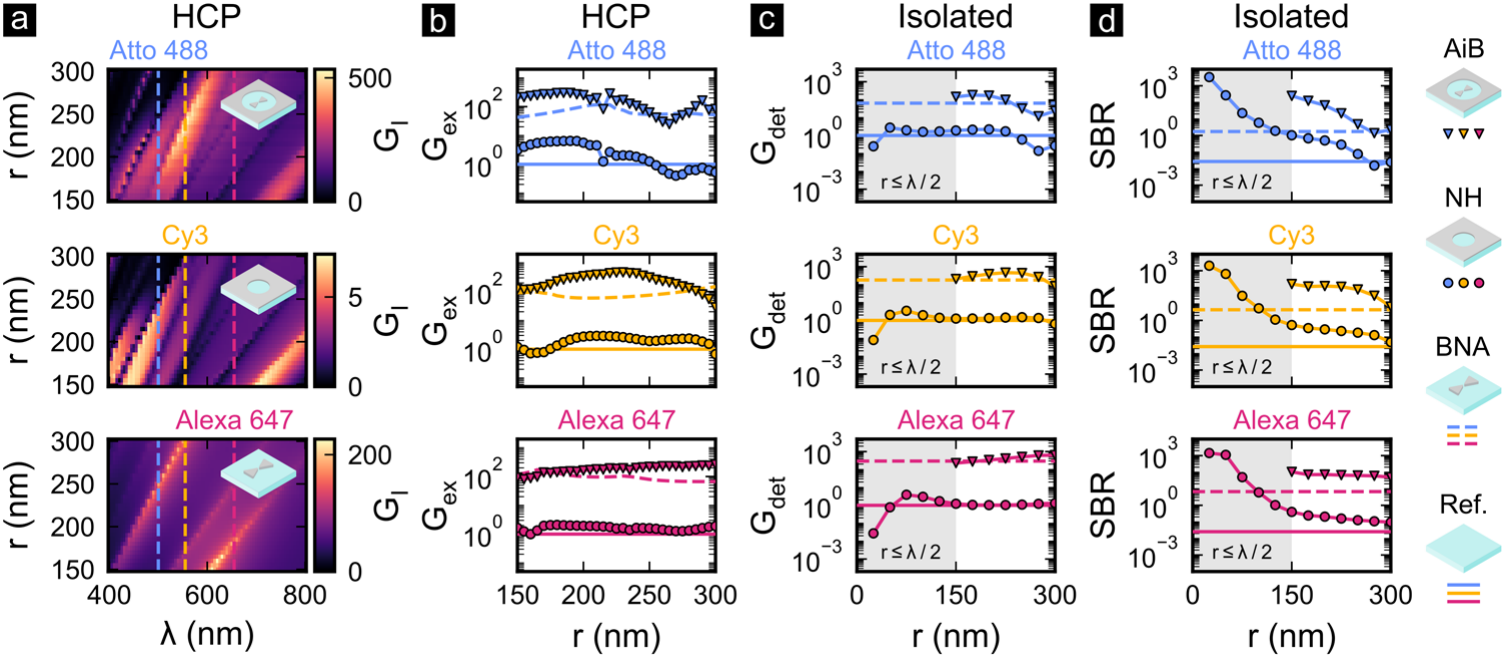
(a) The excitation intensity enhancements *G*_*I*_*(λ)* are computed for HCP-AiBs (top), HCP-NHs (center), and HCP-BNAs (bottom). The vertical dashed lines indicate the maxima of the absorption spectra of Atto 488 (blue), Cy3 (yellow), and Alexa 647 (red). (b) The excitation rate enhancements *G*_ex_ provided by HCP-AiBs (triangular markers), HCP-NHs (round markers), HCP-BNAs (dashed lines) are compared to fluorophores in free space (solid lines). The (c) fluorescence detection rate enhancement *G*_det_ and (d) SBR are computed for (nonperiodic) isolated AiBs, NHs, and BNAs. The gray shaded areas in (c, d) indicate subwavelength radii and the regime where the BNAs no longer physically fit into the NHs. All simulations were carried out for *l* = 80 nm, *h* = 50 nm, *g* = 20 nm, α = 90°, and Δ*r* = 250 nm with a 100 nm PMMA layer on top of a glass (BK7) substrate. The equivalent fluorophore concentrations for the SBR estimations are *c* = 820 nM for all three fluorophores.

Figure 2 (a) shows the excitation intensity enhancement *G*_*I*_*(λ)* in the hotspot of HCP-AiBs, HCP-NHs, and HCP-BNAs for different radii with the maximal absorption wavelengths of the three fluorophores overlaid. The excitation intensity enhancement
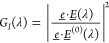
2is calculated by projecting the excitation fields in the hotspot *E̲*(λ) and in free space *E̲*^(0)^(λ) onto the orientation of the fluorophore’s dipole moment *e̲*. We define the hotspot as the center of the BNA gap or NH aperture, respectively. Although HCP-BNAs do not have a nanoaperture and thus no radius *r*, an equivalent radius *r* = *R*-*Δr* can be derived from the half center-to-center distance *R* for better comparability. The excitation intensity enhancement shows a complex modal structure originating from the interplay of the BNA plasmon resonance, the NH cavity modes, and lattice modes from the periodic hexagonal packing. Similar to previous findings, we find that HCP-AiBs provide excitation intensities far above the ones of NHs and even BNAs mostly due to the coupling of the NH cavity mode to the BNA plasmon resonance.^25^ Furthermore, we observe a red shift of the resonances for increasing radii for the three plasmonic systems under study.

Figure 2 (b) shows the excitation rate enhancement *G*_ex_ of HCP-AiBs (triangular markers) compared to the one of HCP-BNAs (dashed lines), HCP-NHs (round markers), and the free space reference (solid lines). In contrast to the excitation intensity enhancement *G*_*I*_(λ), the excitation rate enhancement
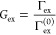
3compares the fluorescence excitation rate in the hotspot Γ_ex_ and in free space Γ_ex_^(0)^ and thus additionally considers the spectral overlap between the excitation source, optical filters, the plasmon resonance, and the fluorophores’ absorption spectrum. Therefore, these simulations indicate at which radii the fluorophores are most efficiently excited. Based on these insights, HCP-AiB radii of *r* = 200–250 nm are found to provide the highest excitation rates for all three fluorophores, exceeding the ones provided by HCP-NHs, HCP-BNAs, and free space.

The excitation rate enhancement helps to identify regimes of efficient fluorescence excitation but does not directly provide regimes of efficient fluorescence emission. This is because the fluorescence emission rate is also influenced by the change of quantum efficiency induced by the Purcell effect.^14^ Therefore, we utilize the fluorescence detection rate enhancement *G*_det_ and the SBR shown in Figure 2 (c) and (d) as two key metrics to assess the single-molecule sensitivity. The fluorescence detection rate enhancement
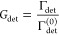
4compares the rate of detected photons Γ_det_ and Γ_det_^(0)^ coming from a fluorophore in a plasmonic hotspot and in free space, respectively. It is closely related to the fluorescence emission rate enhancement experienced by a fluorophore near a plasmonic structure, but additionally accounts for the spectral overlap between the emission spectrum of the fluorophores, the optical filters, and the quantum efficiency of the photodetector. Details on the calculation of the detection rate enhancement and its dependence on the plasmon resonance can be found in Section 2.3 of the Supporting Information. The SBR compares the fluorescence detection rate from a fluorophore in the hotspot (signal) to the cumulated fluorescence detection rate from surrounding fluorophores (background) in a 100 nm thick PMMA layer and at a concentration of *c* = 820 nM. Unfortunately, simulating *G*_det_ and SBR for periodic systems is a nontrivial task so we limit these results to isolated (nonperiodic) AiBs, NHs, and BNAs with otherwise the same geometrical parameters.

The results shown in Figure 2 (c) suggest that an excellent fluorescence detection rate enhancement is provided by isolated AiBs at *r* = 225 nm for all three fluorophores. Remarkably, it outperforms all reference designs in this regime in terms of detection rate enhancement. Expectedly, Figure 2 (d) shows that the highest SBR is offered by isolated AiBs with the smallest radius. At small radii, SBRs well above unity are ensured despite the relatively high concentrations indicating that the SBR should be sufficient for single-molecule studies in the high micromolar regime. Interestingly, the SBR of isolated AiBs is only outperformed by isolated NHs–that are equivalent to ZMWs for subwavelength radii–at *r* ≤ 50 nm because of the strong fluorescence background reduction at such small radii. However, in this regime the fluorescence detection rate of isolated NHs starts dropping below unity suggesting that single-molecule sensitivity might not be ensured anymore. These results underline that AiBs provide an unmatched balance between high *G*_det_ and SBR and highlight the importance of considering both quantities to assess the single-molecule sensitivity of nanophotonic single-molecule sensors. Based on these figures of merit, we decide for an HCP-AiB radius of *r* = 225 nm to achieve an optimal balance between high fluorescence detection rate enhancement and SBR.

**Figure 3:**
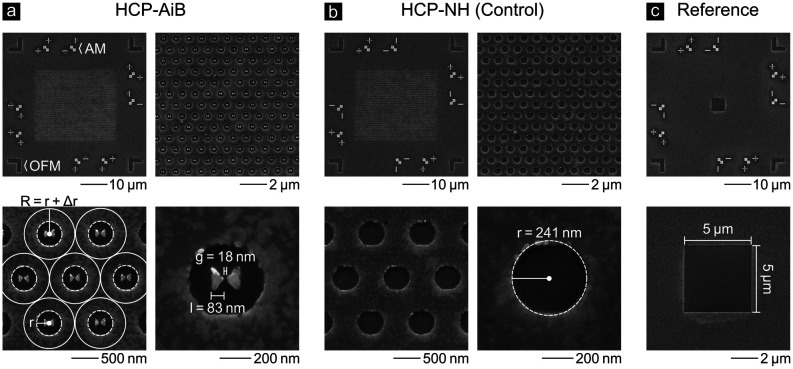
SEM images of the (a) HCP-AiB, (b) HCP-NH, and (c) reference fields. Different magnifications of the same HCP-AiB array are shown in (a). Each array consists of 32 × 32 = 1024 aluminum HCP-AiBs and is surrounded by alignment markers (AMs) and optical fiducial markers (OFMs) required for the fabrication and image analysis, respectively. The NH radii *r* and half center-to-center distances R = *r* + Δ*r* are shown as white dashed and solid lines. The measured BNA length and gap size are *l* = 83 nm and *g* = 18 nm. Their estimated variability is about ±5 nm. (b) HCP-NHs are fabricated with the same geometrical parameters as the HCP-AiBs but without a BNA inside the NH. The NH has a measured radius of *r* = 241 nm. (c) A 5 μm square aperture is used as a reference field and is surrounded by the same OFMs. Each sample contains 25 fields of all three types.

Figure 3 (a) shows scanning electron microscope (SEM) images of a 32 × 32 HCP-AiB array fabricated with a three-step EBL overlay process that is detailed in Figure S3 and associated text of the Supporting Information and derived from an EBL process we reported earlier.^25^ The geometrical parameters of the fabricated HCP-AiBs agree very well with the computationally optimized parameters, except for a slight ∼10% increase of *r* that is still within a good range for multicolor applications. The increased radius originates from the proximity effect that broadens patterns during the EBL exposure. The SEM images further show alignment markers (AMs) used for the EBL overlay process and the OFMs that are important for the optical image registration of the three detection channels. Figure 3 (b) and (c) show an HCP-NH and reference field, respectively, that are both on the same chip as the HCP-AiBs. The HCP-NHs are used as a control to isolate the effect of the BNAs but otherwise have the same geometry as HCP-AiBs. The reference field consists of a 5 μm square aperture that is used to determine the fluorescence detection rate for fluorophores in free space on the same sample and to derive the fluorescence detection rate enhancement provided by HCP-AiBs.

We customized an epi-widefield microscope (see Figure S4) to assess the multicolor single-molecule detection capabilities of HCP-AiBs. The microscope consists of a multichannel LED synchronized with an sCMOS camera such that each frame acquisition triggers a change of the LED excitation channel. This alternating excitation scheme enables to disentangle the three excitation channels on a frame-by-frame basis as illustrated in Figure 1 (b) with a time shift between the channels that corresponds to the exposure time *δt*. Figure S4 (a) shows the transmission spectra of the optical filter set with the LED excitation spectra and fluorophore absorption and emission spectra overlaid. The three excitation and detection channels 1–3 are assigned to Atto 488, Cy3, and Alexa 647, respectively. We conclude from Figure S5 and associated text of the Supporting Information that the fluorescence cross-talk between the channels is low to moderate and had no relevant influence on the single-molecule analyses presented here. The LED excitation light is unpolarized, reducing the HCP-AiB excitation efficiency but rendering rotational misalignment between the polarization and the HCP-AiB arrays irrelevant. For all experiments, the three fluorophores were embedded in an 80 nm thick PMMA layer coating the HCP-AiB, HCP-NH, and reference fields.

Three types of measurements were carried out with this experimental configuration to characterize the HCP-AiB performance. First, epi-widefield imaging was performed at a fluorophore concentration of *c* = 5 μM with a frame rate of *f* = 1.67 fps and an exposure time of *δt* = 200 ms to demonstrate the multicolor single-molecule detection sensitivity at micromolar concentrations. Second, a concentration of *c* = 50 nM was used with the same frame rate and exposure time to allow for single-molecule detection in free space with the reference fields shown in Figure 3 (c). These measurements are important to determine the multicolor fluorescence detection rate enhancement provided by HCP-AiBs. The concentrations correspond to an average number of molecules per hotspot of ⟨*N*⟩ = 0.15 at 5 μM and ⟨*N*⟩ = 0.0015 at *c* = 50 nM with *V* ≈ 20 × 50 × 50 nm^3^. Third, high-speed imaging was carried out at *c* = 50 nM with *f* = 33.33 fps and *δt* = 10 ms to demonstrate that the multicolor single-molecule sensitivity is maintained at high acquisition speeds as required for dynamic single-molecule studies. Exemplary fluorescence images of all channels can be found in Figure S6 for the HCP-AiB, HCP-NH, and reference fields at *c* = 5 μM and *c* = 50 nM.

**Figure 4:**
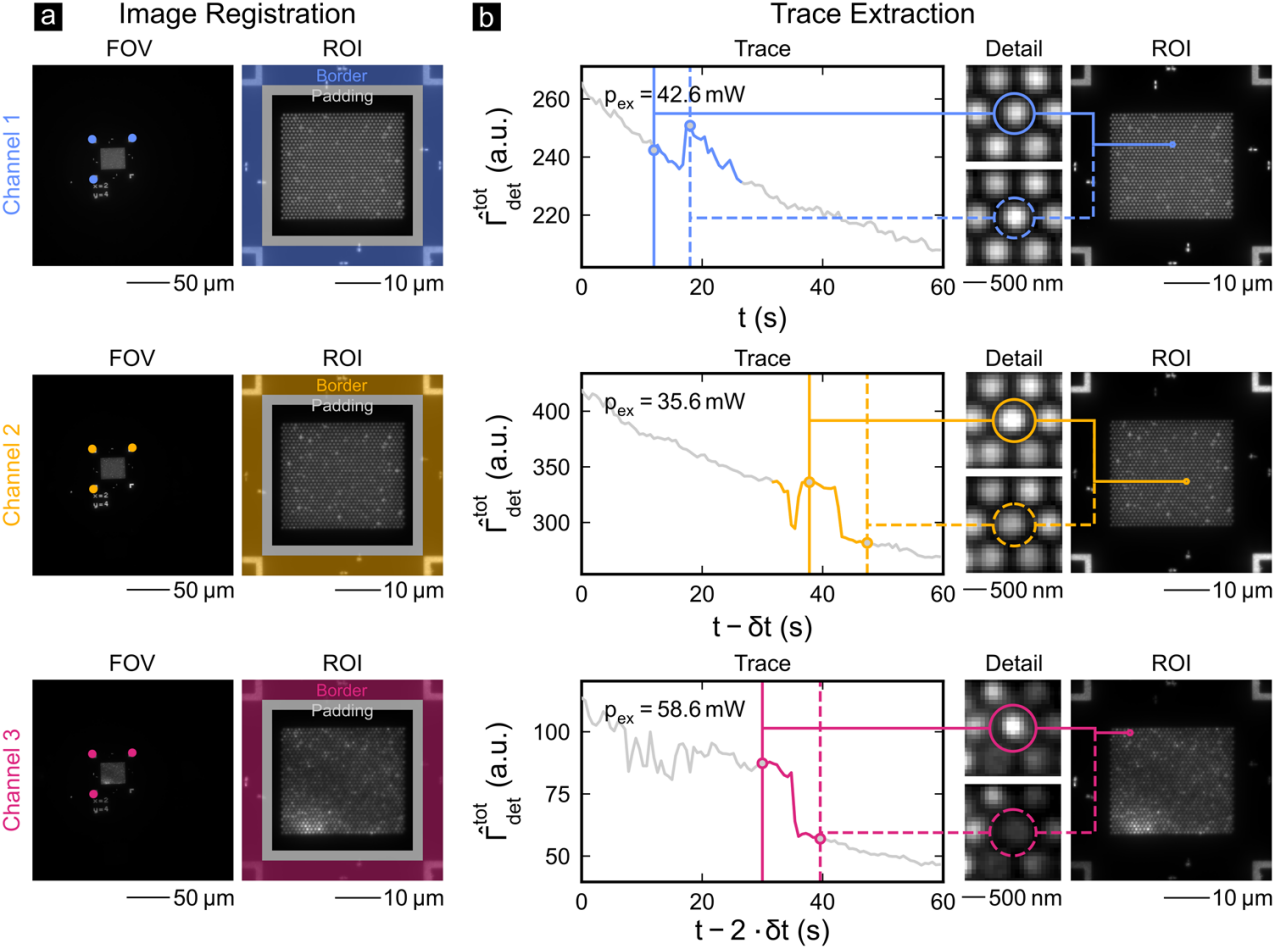
In (a), the sequentially acquired channels 1 (top), 2 (center), and 3 (bottom) are shown for the full FOVs (left) and the automatically registered ROIs (right). Three OFMs are used for the image registration and channel alignment (see round markers in FOVs). Each ROI is split into the border area containing all markers (colored area), an intermediate padding (gray area), and the area containing the HCP-AiB array. (b) Within the latter area each HCP-AiB is detected through an adaptive blob detection algorithm. The fluorescence time traces (gray lines) are the mean signal of each readout area (solid and dashed circles in the detailed views). Blinking and bleaching events are detected automatically in the time traces (colored segments). The solid and dashed vertical lines indicate two time points associated with the detailed views in the central column of (b). The rightmost column of (b) shows the registered ROIs and the HCP-AiBs belonging to the depicted fluorescence time traces. The excitation powers *p*_ex_ are shown on the top left of the time traces. The corresponding excitation power densities for channels 1–3 are 71 W/cm^2^, 59 W/cm^2^, and 98 W/cm^2^(FOV = 245 μm × 245 μm). The images were recorded at *c* = 5 μM and *f* = 1.67 fps (*δt* = 200 ms).

Figure 4 shows how the single-molecule fluorescence detection rate is automatically retrieved from the raw camera frames for all three detection channels. We followed four steps to extract the fluorescence time traces from the camera frames. First, the regions of interest (ROIs, 50 μm × 50 μm) are automatically extracted from the full fields of view (FOVs, 245 μm × 245 μm) using template matching of the OFMs and an affine transformation. This precisely overlays the ROIs of all three detection channels rendering an accurate prealignment of the channels redundant. Second, the aligned ROIs are divided into a border region including all markers, a padding region as a safety margin, and the region including the HCP-AiB arrays. Only in the latter region an adaptive blob detection algorithm (Laplacian of Gaussian) determines the center of each HCP-AiB based on their effective radius *r*_eff_ = *r* + *r*_airy_ and the number of HCP-AiBs per array (i.e., a total of 32 × 32 = 1024). Then, the detected centers and the effective radii are used in a third step to define a readout area in which the mean signal per frame is calculated yielding the normalized total fluorescence detection rate
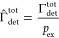
5for each HCP-AiB with Γ_det_^tot^ being the raw total detection rate and *p*_ex_ the excitation power. Finally, the single-molecule fluorescence detection rates Γ̂_det_ are extracted in a fourth step from fluorescence blinking and bleaching events detected in the fluorescence time traces. Figure 4 (b) shows detected blinking and bleaching events as well as detailed views of the corresponding HCP-AiBs at the beginning and the end of such events for all three detection channels. The algorithm used for the automatic detection of blinking and bleaching events is detailed in Figure S7 and associated text of the Supporting Information. The Supporting Information contains videos (S1, S2, and S3) of all three channels recorded simultaneously on an HCP-AiB array at *c* = 5 μM.

**Figure 5:**
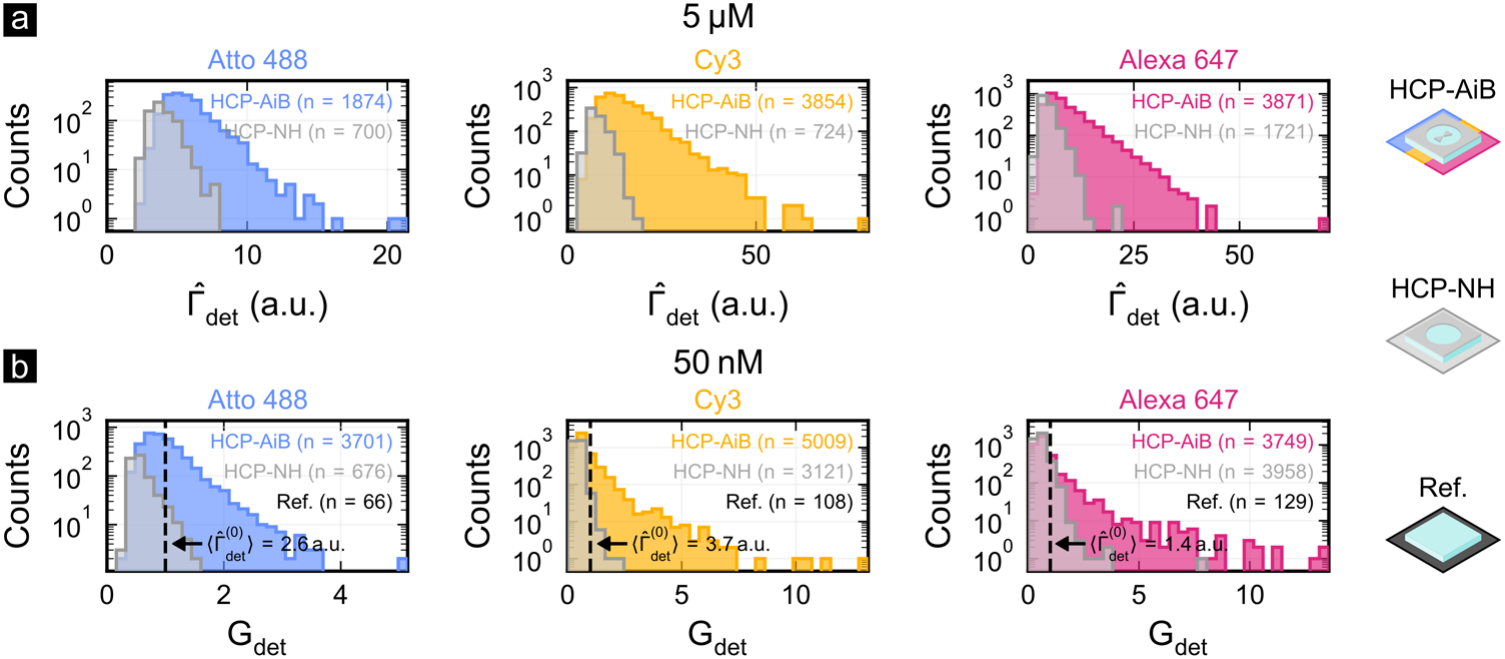
Single-molecule fluorescence (a) detection rate Γ̂_det_ and (b) detection rate enhancement *G*_det_ are determined for Atto 488, Cy3, and Alexa 647 at 5 μM and 50 nM, respectively. The colored histograms belong to HCP-AiBs, the gray histograms to HCP-NHs, and the black dashed line in (b) corresponds to the mean value from the reference measurements ⟨Γ̂_det_^(0)^⟩. *n* is the total number of blinking and bleaching events that were detected to retrieve the single-molecule fluorescence detection rate. The underlying data were recorded for ten fields each and for 60 s at *f* = 1.67 fps (*δt* = 200 ms).

Figure 5 (a) shows histograms of the extracted single-molecule fluorescence detection rates of Atto 488, Cy3, and Alexa 647 at *c* = 5 μM for HCP-AiBs (colored) and HCP-NHs (gray). The histograms are derived from measurements of ten fields each for HCP-AiBs and HCP-NHs. The variability in the histograms is mainly attributed to the random positions and orientations of the fluorophores within the plasmonic hotspot, which influence the degree of enhancement and consequently broaden the histograms. At micromolar concentrations, the reference measurements do not provide single-molecule detection sensitivity preventing the determination of the average reference single-molecule fluorescence detection rate ⟨Γ̂_det_^(0)^⟩ and thus of the experimental single-molecule fluorescence detection rate enhancement
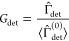
6Nevertheless, these histograms demonstrate that for all three fluorophores HCP-AiBs provide an about 3–4× higher single-molecule fluorescence detection rate (based on the maximal values) and a 2–5× higher number of detected blinking and bleaching events as compared to HCP-NHs. Both observations are strong indications of an enhanced single-molecule detection sensitivity at micromolar concentrations originating from the fluorescence emission rate enhancement and fluorescence background reduction offered by HCP-AiBs.

Figure 5 (b) shows similar histograms for the single-molecule fluorescence detection rate enhancement acquired at *c* = 50 nM from ten fields each for HCP-AiBs, HCP-NHs, and the free space reference. Due to the 80 nm thin PMMA layer the fluorescence background is sufficiently reduced at these concentrations to retrieve the free space single-molecule fluorescence detection rate Γ̂_det_^(0)^ from the reference fields. The histograms show that the HCP-AiBs indeed provide a fluorescence detection rate enhancement *G*_det_ between 5 and 14× for all three fluorophores. Notably, a more than five times enhanced fluorescence detection rate is achieved for Atto 488 despite its high quantum efficiency and its fluorescence absorption and emission in the blue wavelength range (λ_abs_ = 499 nm, λ_em_ = 520 nm, η_fl_^(0)^ = 0.8). This is notable since plasmonic nanoantennas are typically employed for single-color fluorescence enhancement of low quantum yield fluorophores in the red or near-infrared.^15,18−20,30,31^ Importantly, the histograms shown in Figure 5 also demonstrate that highly unlikely events can be captured by reading out over 1000 HCP-AiBs in parallel since even the tails of the distributions are sampled. This makes single-molecule studies at nanomolar concentrations feasible, where nanophotonic sensors typically offer low single-molecule detection probabilities due to their small subdiffraction observation volumes.^6,32,33^ We can quantify the throughput of HCP-AiBs by normalizing the number of detected single-molecule events *n* by the acquisition time (60 s) and number of measured fields (10). This results in a rate of 3–6 and 6–8 single-molecule events per second and field at concentrations of *c* = 5 μM and *c* = 50 nM, respectively. Interestingly, the throughput is higher at lower concentrations as the low fluorescence background allows for higher single-molecule detection sensitivities. This underscores that highly sensitive sensor platforms are also crucial for achieving high throughput.

**Figure 6:**
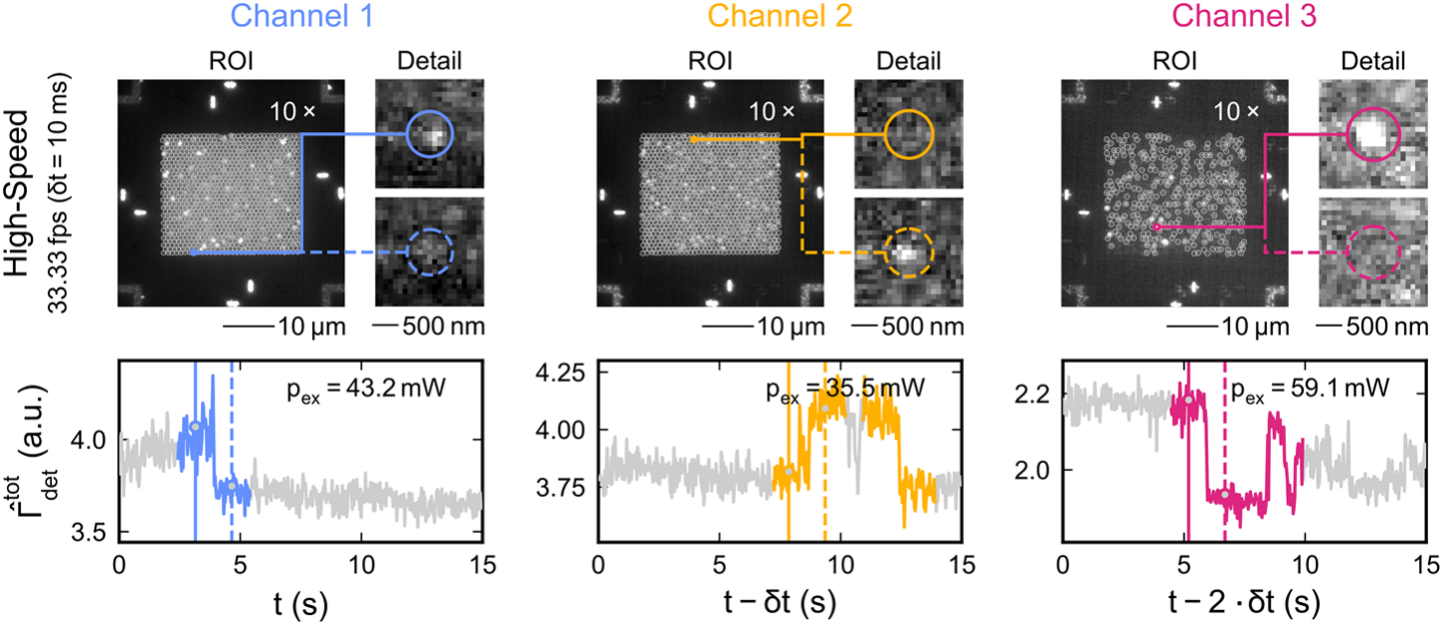
Camera was operated in subarray mode to acquire a reduced FOV with high speed (*f* = 33.33 fps, *δt* = 10 ms). All HCP-AiBs detected within the ROIs are marked by gray circles. The colored circles within the ROIs indicate the HCP-AiBs of which the detailed views and time traces are provided. The ROIs are clipped and brightness adjusted by the value displayed on the top right. The solid and dashed vertical lines in the time traces indicate for each channel the time points for which the two detailed views with the solid and dashed circles are shown. Detected blinking and bleaching events are highlighted as colored segments in the time traces. The excitation powers *p*_ex_ are shown on the top right of the time traces. The corresponding excitation power densities for channels 1–3 are 72 W/cm^2^, 59 W/cm^2^, and 98 W/cm^2^ (FOV = 245 μm × 245 μm). The data were recorded at *c* = 50 nM.

Finally, Figure 6 demonstrates that multicolor single-molecule sensitivity is maintained at high readout speeds of *f* = 33.33 fps and *δt* = 10 ms. To achieve the high-speed imaging, the FOV was reduced to 61 μm × 61 μm by operating the sCMOS in subarray mode. Here, the high HCP-AiB packing density close to the theoretical packing limit imposed by the diffraction limit is crucial as the FOV can be reduced without sacrificing the high throughput. This allows for very high frame rates and manageable amounts of data when long acquisitions are required. Despite the short exposure times and the low fluorophore concentration of *c* = 50 nM, enough signal was acquired to automatically extract the aligned ROIs and fluorescence time traces. At such low concentrations, some HCP-AiBs remained undetected due to the lack of fluorophores in their hotspots and the low fluorescence background. The retrieved fluorescence time traces unambiguously show the blinking and bleaching of fluorophores on all three detection channels and thus demonstrate multicolor single-molecule sensitivity also when imaging at high speeds. Especially for Cy3 and Alexa 647 the high signal-to-noise ratios indicate that readout speeds in the submillisecond range could be feasible. Conquering the micro- to millisecond range is essential to also capture highly transient biological dynamics. For example, the diffusion coefficient of transmembrane receptors within the plasma membrane of cells typically ranges between *D* = 0.01–0.1 μm^2^/s.^34,35^ This implies a dwell time in the HCP-AiB hotspot of τ_*D*_ = Δ*w*^2^/(4·*D*) = 6.25–62.5 ms, assuming a round hotspot of width Δ*w* = 50 nm. Therefore, our results indicate that high-throughput multicolor cross-correlation studies even of highly transient dynamics are within reach with the high fluorescence detection rate enhancement and fluorescence background reduction offered by HCP-AiB arrays.

## 3 Conclusion

In this work, we demonstrated that aluminum-based high-density HCP-AiBs deliver three key advancements to the field of nanophotonic biosensors for single-molecule studies. First, we showed that HCP-AiBs provide multicolor single-molecule sensitivity at micromolar concentrations of the widely used fluorophores Atto 488, Cy3, and Alexa 647 without the use of chemical quenchers. Second, we illustrated how combining high-density HCP-AiBs with OFMs allows for the parallel readout of over 1000 HCP-AiBs enabling high-throughput and alignment-free multicolor cross-correlation studies. The packing density ρ_hcp_ of the HCP-AiBs arrays reported here (2·*R*_hcp_ = 2·R = 950 nm) is
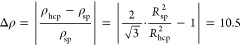
7times higher as compared to the packing density ρ_sp_ of previous square-packed AiB platforms with a periodicity of about 2·*R*_sp_ = 3 μm.^24,25^ The high packing density also facilitated the detection of single molecules down to nanomolar concentrations, which is typically complicated by the small subdiffraction observation volumes of nanophotonic sensors. Third, we showed that the multicolor single-molecule sensitivity is maintained even at exposure times in the low millisecond range. This is mainly enabled by the multicolor fluorescence detection rate enhancement provided by HCP-AiBs and is crucial to capture highly transient single-molecule dynamics in biological systems.

The overall performance of the HCP-AiB arrays was achieved through the careful computational optimization of their geometry and the development of an EBL overlay process with sufficient resolution. For the computational optimization we employed the fluorescence detection rate enhancement *G*_det_ and the SBR as the two main performance metrics to quantify the single-molecule sensitivity. Based on these metrics, we showed that aluminum AiBs provide a combination of very high *G*_det_ and SBR that is unmatched by other common types of nanophotonic single-molecule sensors. We identified the radii *r* = 200–250 nm as the optimal range to establish a good balance between high SBR and *G*_det_ for multicolor applications and showed that our fabrication process yields high-density HCP-AiBs with the desired geometrical parameters.

We used an epi-widefield microscope with a sequential three-color excitation scheme and a camera-based detection to experimentally verify the HCP-AiB capabilities. These experiments confirmed the single-molecule sensitivity at micromolar concentrations and the multicolor fluorescence detection rate enhancement. We observed multicolor fluorescence detection rate enhancement factors of 5 for Atto 488, 13 for Cy3, and 14 for Alexa 647. Comparable studies have reported enhancement factors of 15–30 for fluorophores like Atto 488 and up to 50 for Alexa 647.^25,36,37^ However, these studies were conducted with single-color detection, allowing for optimization of the plasmon resonance within a narrow spectral range. A detailed discussion on comparing enhancement factors across studies is provided in Section 7 of the Supporting Information. We attribute the discrepancy between our experimental and simulated fluorescence detection rate enhancement to three main factors. First, the spatial discretization in our simulations causes an overestimation of the enhancement factors due to staircasing. Second, the experiments are performed with unpolarized and high-NA illumination, in contrast to the linearly polarized plane wave illumination in the simulations. Third, the simulations use refractive index data for pristine materials, but real-world issues like limited fabrication resolution, material granularity, oxidation, and geometric asymmetries degrade the material and thus the resonance quality.

The sequential excitation scheme allows a high number of excitation channels with a single camera but requires exposure times well below the typical time scales of the observed dynamics to ensure quasi-simultaneous multicolor readout for cross-correlation studies. Because of this, capturing even the most short-lived single-molecule dynamics requires exposure times in the upper microsecond range. This is not out of reach with modern sCMOS cameras that can provide such exposure times when limiting the number of readout lines. For example, the camera used here provides frame rates of *f* ≈ 2340 fps (assuming three channels) and exposure times of *δt* ≈ 140 μs when exposing only 28 × 2304 of the 2304 × 2304 pixels. At the current magnification (60 × ) this would allow to maintain the number of HCP-AiBs per FOV using a 4 × 256 = 1024 instead of the current 32 × 32 = 1024 configuration of the arrays. Here, the almost 11 times higher packing density of the HCP-AiBs provides a critical advantage in enabling such high readout speeds without sacrificing throughput.

Although the employed fabrication process provides very high flexibility in terms of the HCP-AiB design and excellent resolution, its complexity remains a bottleneck in making HCP-AiBs widely available for multicolor single-molecule studies. The most promising approach to reduce the complexity of the lithography process is the combination of an etch-resistant positive-tone resist and reactive-ion etching (RIE) with chlorine-based etchants.^38^ In addition, emerging technologies like ion beam lithography (IBL) could also facilitate the fabrication of high-density HCP-AiB arrays.^39^ Enabling the fabrication of HCP-AiBs with a single lithography step would make HCP-AiBs as cost-effective as many other lithography-based nanophotonic platforms. If high cost-effectiveness is a primary requirement, one could employ colloidal lithography to fabricate high-density ZMW arrays.^40^ Despite their significantly reduced fluorescence enhancement factors, aluminum-based ZMWs could provide a low-cost alternative to HCP-AiBs that also benefits from the analysis pipeline presented here.

As shown in this study, high-density HCP-AiB arrays excel when both high fluorescence enhancement and low fluorescence background are required to provide high single-molecule detection sensitivity. This is crucial when capturing dynamics at micro- to millisecond time scales or working with weakly fluorescent molecules. In this regard, HCP-AiBs combine the unique abilities of simultaneously providing fluorescence background reduction and fluorescence enhancement to work equally with strongly and weakly fluorescent molecules. This allows for a much broader palette of available fluorophores and thus more experimental flexibility.

Equipped with these capabilities, HCP-AiBs now need to showcase them in real-world biosensing applications.^41−43^ This will require the exploration of new surface functionalization protocols and protective measures to increase the biocompatibility.^44^ The passivation of aluminum with ultrathin Al_2_O_3_ or SiO_2_ layers has been shown to improve the stability of plasmonic nanostructures and to protect biological specimens from undesired interactions with the plasmonic material.^9,29^ Furthermore, an increasing body of nanophotonic biosensors relies on the use of aluminum instead of gold due to its superior opaqueness, broadband plasmon resonances down to the ultraviolet wavelength range, and compatibility with CMOS processes.^33,45,46^ Therefore, new surface functionalization protocols can be expected to benefit the field of nanophotonic biosensors as a whole and not solely HCP-AiBs.

## 4 Materials and Methods

The epi-widefield images are taken with an sCMOS camera (ORCA-Fusion BT, Hamamatsu) and a quad-channel LED (pE-400^max^ MB, CoolLED) attached to an Olympus IX71 inverted microscopy body. The sCMOS camera is connected to the LED with a coaxial cable (CA2848, Thorlabs) and triggers with each frame acquisition the next excitation channel in a predefined sequence via a transistor-transistor logic (TTL) signal. A quad-band filter set (89402 - ET – 391–32/479–33/554–24/638–31 multi LED set, Chroma) is used to allow for up to four excitation and detection channels. The light is focused on and collected from the sample with an Olympus UPlanSApo 60 × , NA = 1.2 water-immersion objective. The excitation power is measured for each channel after the objective with a power meter (PM100D and S120C, Thorlabs) at the center wavelength of the corresponding transmission window of the excitation filter. The sample contained 25 HCP-AiB fields, 25 HCP-NH fields, and 25 reference fields. Exemplary fields of each type are shown in Figure S6 at (a) *c* = 5 μM and (b) *c* = 50 nM. For each type of field, we measured ten distinct fields for 60 s at *f* = 1.67 fps (i.e., 30 total acquisitions at *c* = 5 μM and 30 total acquisitions at *c* = 50 nM) and five distinct fields for 15 sat *f* = 33.33 fps (i.e., 15 total acquisitions at *c* = 50 nM). The fluorophores Atto 488 (Atto 488 carboxylic acid, ATTO-TEC), Cy3 (Cyanine3 carboxylic acid, Lumiprobe), and Alexa 647 (AF 647 carboxylic acid, Lumiprobe) were diluted in PMMA (AR-P639.04, Allresist) to *c* = 5 μM and *c* = 50 nM, resulting in a total fluorophore concentration of *c* = 15 μM and *c* = 150 nM, respectively. The sample was then prepared by spin-coating it with the doped PMMA for 1 min at 4000 rpm and finally baking it for 3 min at 155 °C resulting in a 80 nm thick PMMA layer on top of the sample. The backside of the sample was thoroughly cleaned with acetone to remove any PMMA residues.

## Supporting Information Available

Preliminary FDTD simulations of the optimal HCP-AiB design, detailed discussion of the enhancement factor simulations, explanation of the HCP-AiB fabrication, explanation of the sequential multicolor widefield microscope, analysis of the fluorescence cross-talk, discussion of the fluorescence time trace analysis, discussion of important considerations for comparing fluorescence enhancement factors (PDF)
Video of channel 1 (Atto 488) recorded simultaneously with the other two channels on an HCP-AiB array (MP4)
Video of channel 2 (Cy3) recorded simultaneously with the other two channels on an HCP-AiB array (MP4)
Video of channel 3 (Alexa 647) recorded simultaneously with the other two channels on an HCP-AiB array (MP4)
