# Supplementary material for: Hexagonal Plasmonic Arrays for High-Throughput Multicolor Single-Molecule Studies
Source: ACS Appl Mater Interfaces. 2024 Jul 23;16(31):41271–80. doi: 10.1021/acsami.4c04744 (PMC11310910; doi:10.1021/acsami.4c04744)
Supplement: Supplementary file 1 — am4c04744_si_001.pdf [file am4c04744_si_001.pdf]

Supporting Information

Hexagonal Plasmonic Arrays for High-Throughput Multicolor Single-Molecule Studies

Ediz Kaan Herkert[1,*], *Lukas Lau*[1], *Roger Pons Lanau*[1], *Maria F. Garcia-Parajo*[1,2]

[1] ICFO - Institut de Ciencies Fotoniques, The Barcelona Institute of Science and Technology, 08860, Castelldefels (Barcelona), Spain

[2] ICREA, 08010, Barcelona, Spain

[*]Corresponding Author

Name: Ediz Kaan Herkert

E-Mail: ediz.herkert@icfo.eu

Mobile: +34 643 712 243

ORCID: 0000-0003-3040-8077

Name: Lukas Lau

E-Mail: lukas.lau@icfo.eu

ORCID: 0000-0002-4780-524X

Name: Roger Pons Lanau

E-Mail: roger.pons@icfo.eu

ORCID: 0000-0001-6666-2435

Name: Maria F. Garcia-Parajo

E-Mail: maria.garcia-parajo@icfo.eu

ORCID: 0000-0001-6618-3944

1. Preliminary HCP-AiB Design Optimization

As pointed out in the main text, the HCP-AiBs design is described by a large number of geometrical parameters. The BNAs are described by their length l, gap size g, and apex angle α, the NHs by their radius r, and the HCP arrangement by the half center-to-center distance $R = r + \Delta r$ with Δr being the half edge-to-edge distance. Furthermore, the aluminum film has a thickness h. Rigorously optimizing all seven parameters is computationally very expensive but due to fabrication constraints it is possible to reduce the number of feasible parameters.

Theoretically, the enhancement provided by nanoantennas increases with smaller gap sizes until about $g = 10\ \mathrm{nm}$, beyond which fluorescence quenching mechanisms begin reducing the fluorophore emission.[1] We select $g = 20$ nm for our HCP-AiB design, as this is the smallest gap size that can be reliably fabricated with the EBL process used here.

The thickness h of the aluminum film can also be derived from theoretical considerations and fabrication limitations. Typically, plasmon resonances shift towards the red with decreasing nanoantenna heights.[2] To maintain the plasmon resonance within a desired spectral region, a smaller height h needs to be compensated by a smaller length l. However, reducing the lateral size (here the length l) can result in a loss of structural features due to the limited fabrication resolution. This imposes a lower limit on the height h below which too many lateral features of the nanoantenna are lost. Additionally, low heights introduce more interfacial damping, which reduces achievable enhancement factors.[2] The upper height limit is set by the thickness of the electron beam resist used during the EBL fabrication process. In lift-off processes, the thickness of the deposited metal must be kept well below the resist thickness, which is 80 nm for the process used here. Because of this, a height of $h = 50$ nm provides a good compromise between a reliable lift-off and a sufficient lateral size.

This limits the parameters to the length l, apex angle α, and radius r, as the half center-to-center distance R is constrained by r and Δr, with the latter limited to 250 nm by the diffraction limit. We optimize these three parameters based on the excitation rate enhancement G_{ex} using a coarse grid search. The excitation rate enhancement is chosen as objective function as it is computationally less expensive than the detection rate enhancement and SBR, allowing to search a larger parameter space. The data in Figure S1 show that an apex angle of $\alpha = 90°$ and a length of $l = 80$ nm yield a high excitation rate enhancement that is well-balanced across all fluorophores. For Alexa 647, the optimal parameters lie outside of the simulated parameter space so that the values shown in Figure S1 do not indicate the global maxima.

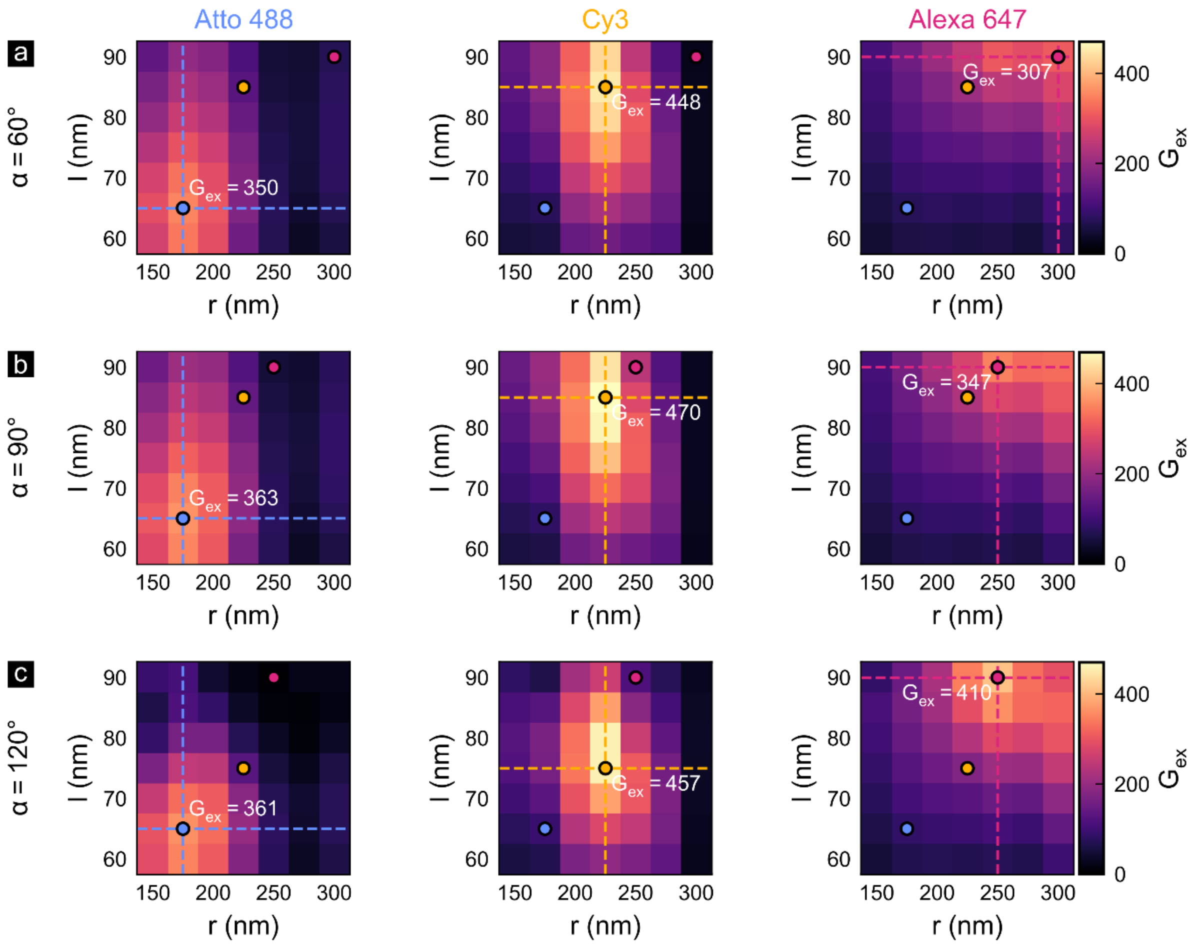

Figure S1. The excitation rate enhancement G_{ex} was used to optimize various HCP-AiB design parameters. Local maxima of the excitation rate are indicated by colored markers for Atto 488 (blue), Cy3 (yellow), and Alexa 647 (red) and (a) $\alpha = 60°$, (b) $\alpha = 90°$, and (c) $\alpha = 120°$. This allows to determine lengths l, radii r, and apex angles α that provide high excitation rates across all three fluorophores.

Based on these considerations, we choose $l = 80$ nm, $g = 20$ nm, $\alpha = 90°$, $\Delta r = 250$ nm, and $h = 50$ nm. The radius r is further refined in the main text through comprehensive optimization since it is the only parameter that influences the emission rate enhancement, the signal-to-background ratio, and cavity and lattice modes.

2. Simulation of the Enhancement Factors

All simulation results presented here are obtained with the commercially available finite-difference time-domain (FDTD) software Lumerical. Periodic hexagonal close-packed (HCP) arrangements were simulated using periodic boundary conditions (PBCs) and a plane wave source. Non-periodic simulations of isolated structures were carried out with perfectly matched layers (PMLs) and a total-field scattered-field (TFSF) source. The excitation fields are always injected from the bottom and are y-polarized along the long axis of the bowtie nanoantenna (BNA).

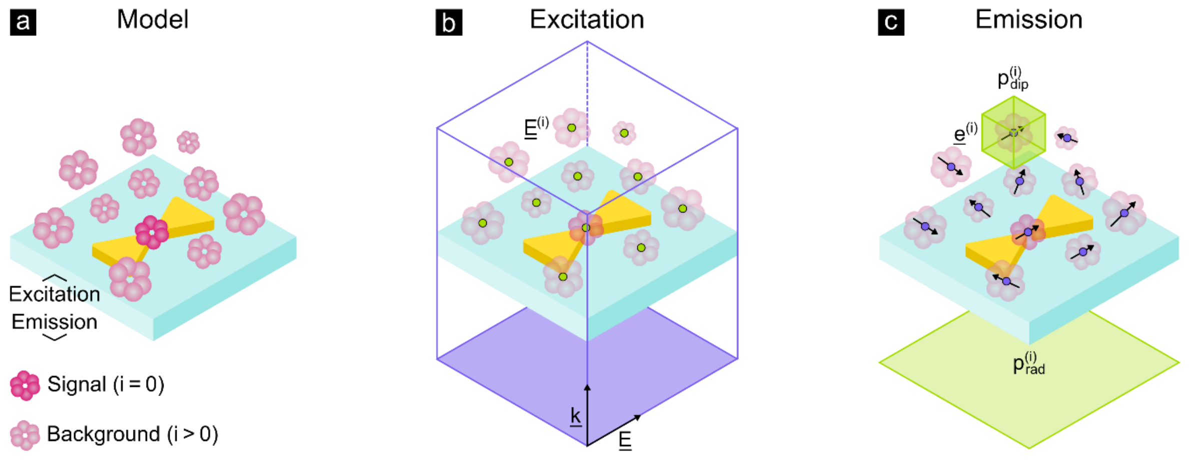

Figure S2. (a) The experimental reality can be modeled by a random distribution of fluorophores and an epi-detection scheme. The fluorophore in the hotspot is the "signal" fluorophore ($i = 0$) and the remaining fluorophores are the "background" fluorophores ($i > 0$). (b) Based on this model the electric field and thus the excitation rate can be calculated for all fluorophores at their respective location using 0D field monitors. For this, a y-polarized plane wave is injected from the bottom (purple plane). (c) The fluorophores are approximated by dipole sources with orientation $\underline{e}$ to record the total emitted power $p_{\mathrm{dip}}^{(i)}$ and the power radiated into the far-field $p_{\mathrm{rad}}^{(i)}$ with transmission monitors (green planes). This allows to calculate the SBR, the fluorescence emission rate enhancement, and consequently the fluorescence detection rate enhancement.

2.1. Excitation Intensity Enhancement

The excitation intensity enhancement is calculated according to Equation (2) of the main text. For this, a 0D monitor is placed in the center of the BNA gap region to record the electric fields $\underline{E}^{(0)}(\lambda)$ in free space (only the BK7 coverslip and PMMA layer) and $\underline{E}(\lambda)$ in the presence of the plasmonic structures (HCP-AiB, HCP-NH, HCP-BNA) for different excitation wavelengths λ. The orientation of the fluorophore's dipole moment $\underline{e}$ is assumed to be along the long BNA axis for most efficient excitation.

2.2. Excitation Rate Enhancement

Based on the simulations performed for the excitation intensity enhancement it is possible to compute the excitation rate enhancement that additionally considers the spectral overlap between the excitation source spectrum, the optical filters, the plasmon resonance, and the fluorophore's absorption spectrum. The power absorbed by a fluorophore is

$$p_{\mathrm{abs}}(\lambda) = \sigma_{\mathrm{abs}}(\lambda) \cdot I_{\mathrm{ex}}(\lambda), \qquad \text{(S1)}$$

with $\sigma_{\mathrm{abs}}(\lambda)$ being its absorption cross section and $I_{\mathrm{ex}}(\lambda) = \left|\underline{e} \cdot \underline{E}(\lambda)\right|^2$ the excitation intensity at the fluorophore's location. From the absorbed power the excitation rate is obtained by integrating over the absorbed power

$$\Gamma_{\mathrm{ex}} = \int \frac{p_{\mathrm{abs}}(\lambda)}{h \cdot c} \cdot \lambda \, \mathrm{d}\lambda, \qquad \text{(S2)}$$

with the speed of light c and the Planck constant h. Finally, the excitation intensity $I_{\text{ex}}(\lambda)$ is obtained by considering the spectra of the excitation source $I_{\text{src}}(\lambda)$, the excitation filter $T_{\text{ex}}(\lambda)$, the dichroic filter $T_{\text{di}}(\lambda)$, and the previously calculated excitation intensity enhancement $G_I(\lambda)$

$$I_{\text{ex}}(\lambda) = I_{\text{src}}(\lambda) \cdot T_{\text{ex}}(\lambda) \cdot \left(1 - T_{\text{di}}(\lambda)\right) \cdot G_I(\lambda). \tag{S3}$$

While this does not yield absolute excitation rates, it allows to calculate the excitation rate enhancement according to Equation (3) of the main text.

2.3. Fluorescence Detection Rate Enhancement and Signal-to-Background Ratio

Figure S2 illustrates the considerations involved to calculate the fluorescence detection rate enhancement and SBR. As shown in the model depicted in Figure S2 (a), the fluorophores are randomly distributed in a 100 nm thick PMMA layer at an equivalent concentration of $c = 820\ \text{nM}$. Only one fluorophore – the "signal" fluorophore – is placed in the gap region with its dipole moment aligned with the long BNA axis. The other fluorophores are randomly aligned and positioned and referred to as "background" fluorophores. The "signal" fluorophore is assigned to the index $i = 0$ and the "background" fluorophores to the indices $i > 0$. This allows to calculate the electric fields at each fluorophore location $\underline{E}^{(i)}(\lambda)$ as illustrated in Figure S2 (b) and consequently the excitation rates for each fluorophore $\Gamma_{\text{ex}}^{(i)}$ as described in the previous section. The emission rates then follow from the excitation rates and quantum efficiencies $\eta_{\text{fl}}^{(i)}$

$$\Gamma_{\text{em}}^{(i)} = \Gamma_{\text{ex}}^{(i)} \cdot \eta_{\text{fl}}^{(i)} = \Gamma_{\text{ex}}^{(i)} \cdot \frac{\Gamma_{\text{rad}}^{(i)}}{\Gamma_{\text{rad}}^{(i)} + \Gamma_{\text{nr}}^{(i)} + \Gamma_{\text{loss}}^{(i)}}. \tag{S4}$$

The radiative decay rates $\Gamma_{\text{rad}}^{(i)}$, non-radiative decay rates $\Gamma_{\text{nr}}^{(i)}$, and absorption loss rates $\Gamma_{\text{loss}}^{(i)}$ (induced by ohmic losses of the metal) required to compute the quantum efficiencies of the fluorophores are obtained from a second set of simulations described in Figure S2 (c). Here, dipole sources are positioned and aligned according to the fluorophore locations and orientations. The total power emitted by the dipoles $p_{\text{dip}}^{(i)}$ is then measured using a transmission box surrounding the dipoles measuring the net power passing through it. The power $p_{\text{rad}}^{(i)}$ that is radiated into the far-field (up to almost 90° in epi-direction) is measured with an underlying transmission monitor allowing to calculate the absorbed power

$$p_{\text{loss}}^{(i)} = p_{\text{dip}}^{(i)} - p_{\text{rad}}^{(i)}. \tag{S5}$$

The power lost through non-radiative decays can be approximated from the intrinsic (free space) quantum efficiency $\eta_{\text{fl}}^{(0)}$

$$p_{\text{nr}}^{(i)} = p_{\text{nr}}^{(0,i)} = p_{\text{rad}}^{(0,i)} \cdot \frac{\left(1 - \eta_{\text{fl}}^{(0)}\right)}{\eta_{\text{fl}}^{(0)}}, \tag{S6}$$

assuming that there are no absorption losses in the absence of plasmonic structures and that the latter do not affect the non-radiative decay rates.[3] Here, $p_{\mathrm{nr}}^{(0,i)}$ and $p_{\mathrm{rad}}^{(0,i)}$ are the non-radiative and radiative power losses in free space. The latter is obtained by simulating the power radiated by a dipole source in free space. Knowing the radiative, non-radiative, and absorption power losses $p_{\mathrm{rad}}^{(i)}$, $p_{\mathrm{nr}}^{(i)}$, and $p_{\mathrm{loss}}^{(i)}$, the modified quantum efficiency is then

$$\eta_{\mathrm{fl}}^{(i)} = \frac{p_{\mathrm{rad}}^{(i)}}{p_{\mathrm{rad}}^{(i)}+p_{\mathrm{nr}}^{(i)}+p_{\mathrm{loss}}^{(i)}}, \tag{S7}$$

so that the fluorescence emission rates $\Gamma_{\mathrm{em}}^{(i)}$ can be calculated according to Equation (S4). The fluorescence detection rate – that is the rate at which fluorescence photons are registered by the photodetector – is then calculated by

$$\Gamma_{\mathrm{det}}^{(i)} = \Gamma_{\mathrm{em}}^{(i)} \cdot \int f_{\mathrm{em}}(\lambda) \cdot T_{\mathrm{di}}(\lambda) \cdot T_{\mathrm{em}}(\lambda) \cdot \eta_{\mathrm{det}}(\lambda)\, \mathrm{d}\lambda, \tag{S8}$$

with the fluorophore emission spectrum $f_{\mathrm{em}}(\lambda)$, emission filter $T_{\mathrm{em}}(\lambda)$, and detector quantum efficiency $\eta_{\mathrm{det}}(\lambda)$. The emission spectrum of the fluorophore is assumed to stay unaffected by the plasmonic structures in this model. By performing these simulations in free space and with the plasmonic structures, the detection rate enhancement of the "signal" fluorophore ($i = 0$)

$$G_{\mathrm{det}}^{(i=0)} = \Gamma_{\mathrm{det}}^{(i=0)} / \Gamma_{\mathrm{det}}^{(0,i=0)}, \tag{S9}$$

is obtained according to Equation (4) of the main text. Also, the SBR can be estimated from these computations by dividing the fluorescence detection rate of the "signal" fluorophore ($i = 0$) by the total fluorescence detection rate of the "background" fluorophores ($i > 0$)

$$\mathrm{SBR} = \Gamma_{\mathrm{det}}^{(i=0)} / \sum_{i>0} \Gamma_{\mathrm{det}}^{(i)}. \tag{S10}$$

3. Fabrication of HCP-AiBs

The process used for the fabrication of HCP-AiBs, HCP-NHs, and reference fields is shown in Figure S3. It is based on a three-step EBL overlay process that consists of the fabrication of (i) alignment markers, (ii) the HCP-NH arrays, and (iii) the HCP-BNA arrays. This fabrication process builds upon the process used for the fabrication of hybrid AiBs but requires an additional step to also separate the fabrication of the gold alignment markers and the aluminum BNAs.[4] Therefore, the following description closely follows the previously reported one.

Initially, borosilicate-crown glass (BK7) coverslips (#1.5) were cleaned by 15 min sonication in acetone followed by rigorous rinsing with isopropanol and Milli-Q water. Subsequently, the coverslips were nitrogen blow-dried and put on a hot plate for 3 min at 155°C.

For the first EBL step, the positive-tone resist AR-P6200.04 (Allresist) was spin-coated for 1 min at 4000 rpm and then baked on a hot plate for 2 min at 155°C. The conductive polymer

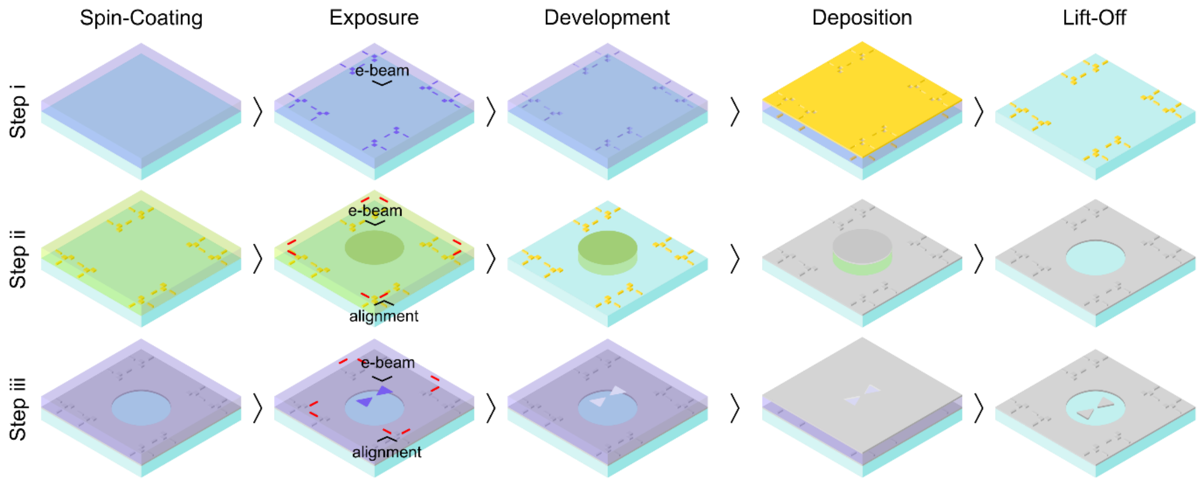

Figure S3. The fabrication of HCP-AiBs is based on three EBL overlay-processes. In step i, a positive-tone lift-off process is used to fabricate two sets of eight alignment markers that are required for the overlay of the following two steps. Steps ii and iii also rely on a lift-off process with a negative- and positive-tone resist, respectively, and separate the NH and BNA fabrication. Both steps employ an automatic alignment procedure to ensure an accurate positioning of the BNAs in the NHs. For this, the eight alignment markers are scanned by the electron beam (red lines) before each exposure to determine the sample coordinate system. Steps ii and iii employ distinct sets of alignment markers as the alignment marker scan leaves adverse resist debris on them.

Espacer 300Z (Showa-Denko) was spin-coated for 1 min at 5000 rpm on top of the resist to prevent charging due to the insulating substrate. For the exposure, a 30 kV Raith Elphy Plus system was used with a small electron beam aperture (30 µm, spot size 2), a step size of 10 nm, and an exposure dose of 162.5 $\mu C/cm^2$. After the exposure, the conductive polymer was removed by a 15 s bath in Milli-Q water and nitrogen blow-drying. The resist was then developed for 2 min in AR 600-546 (Allresist) and rigorously rinsed with isopropanol and Milli-Q water before nitrogen blow-drying. A 3 nm chromium adhesion layer followed by a 50 nm gold layer were deposited using a Leybold Univex 350 evaporator. The lift-off was done with an approximately 2.5 h bath in AR 600-71 (Allresist) followed by 30 min sonication. Afterwards, the sample was immediately rinsed with isopropanol and Milli-Q water and nitrogen blow-dried, concluding the alignment marker fabrication.

Before the second EBL step, the coverslips were baked on a hot plate for about 3 min at 155°C for better resist adhesion. The negative-tone resist AR-N7520.073 (Allresist) was then spin-coated for 1 min at 2000 rpm and baked on a hot plate for 2 min at 100°C. The conductive polymer was spin-coated as detailed above. The NH patterns were exposed with the same EBL system and exposure parameters of the first step but with an exposure dose of 375 $\mu C/cm^2$ for the NH and reference fields and 450 $\mu C/cm^2$ for labels. An automatic alignment procedure detecting the center coordinate of eight markers (four to detect the x- and y-positions, respectively) was used to fully automate the exposure and overlay alignment process. After the exposure, the conductive polymer was removed as detailed before. The resist was then

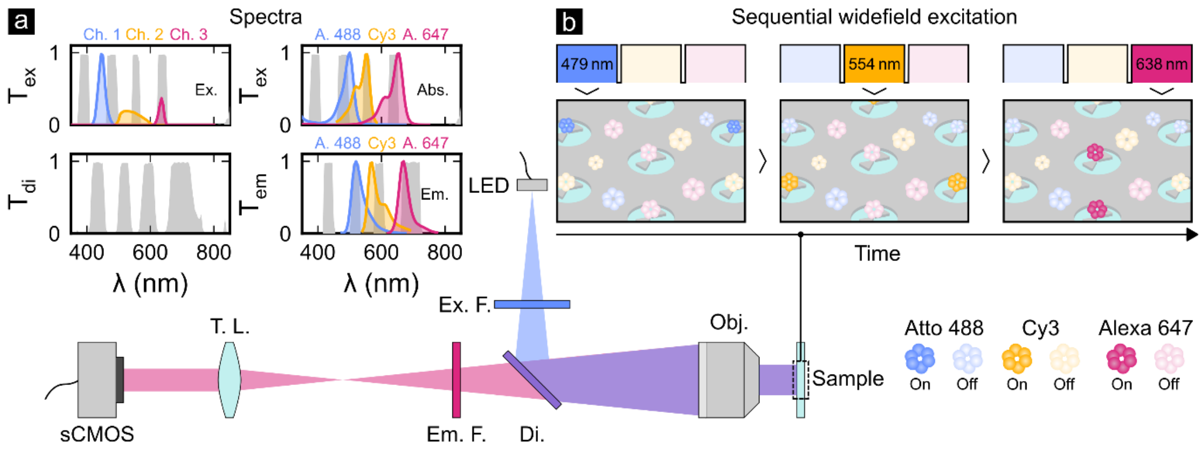

Figure S4. (a) Transmission spectra of the optical filters and (b) sequential widefield excitation scheme. (a) The optical filter set consists of an excitation filter (T_{ex}), dichroic (T_{di}), and an emission filter (T_{em}). The excitation spectra of the four LED channels and the absorption spectra of the fluorophores are shown on top of T_{ex}. The emission spectra of the fluorophores are superimposed with the transmission spectrum of the emission filter T_{em}. (b) Channels 1 – 3 are sequentially exciting the HCP-AiB arrays allowing to temporally separate the signal coming from the three different fluorophores. The spectra are obtained from the manufacturers' website. (T. L.: Tube Lens, Obj.: Objective, Ex. F.: Excitation Filter, Em. F.: Emission Filter, Di.: Dichroic).

developed for 2 min in AR 300-26 (Allresist) with a 1:2 dilution in Milli-Q water. The development was stopped by rinsing with Milli-Q water before nitrogen blow-drying. A 50 nm thick aluminum layer was deposited using the same evaporator as before. The lift-off was done with an immediate 30 min sonication in AR 600-71 (Allresist). Immediately starting the sonication after immersing the sample in the solvent is crucial for a successful lift-off. Afterwards, the sample was rinsed with isopropanol and Milli-Q water and nitrogen blow-dried. For the third EBL step, the same sample preparation protocol, positive-tone resist, and EBL system as in the first step were used. However, a step size of 5 nm was selected to sufficiently sample the smaller BNA features and the automatic alignment process of step ii was performed to accurately position the BNAs within the NHs. Here, a second set of alignment markers was used to avoid the debris on the first set caused by the first alignment procedure in step ii. An exposure dose of 390 $\mu C/cm^2$ yielded the best results for the desired BNA size. After the exposure, the conductive polymer was removed and the resist was developed as described in the first step. After the electron beam exposure, a 50 nm thick aluminum layer was deposited with the same evaporator used previously. For the lift-off, the sample was first immersed 1.5 h and then sonicated 30 min in the same solvent as in step i. The BNA fabrication was finished by cleaning the sample as previously described.

4. Sequential Multicolor Widefield Excitation

Figure S4 shows the transmission spectra of the optical filter set and a sketch of the most relevant optical components of the inverted epi-fluorescence microscope used in this study. In Figure S4 (a), the four excitation channels of the LED source and the absorption spectra of the

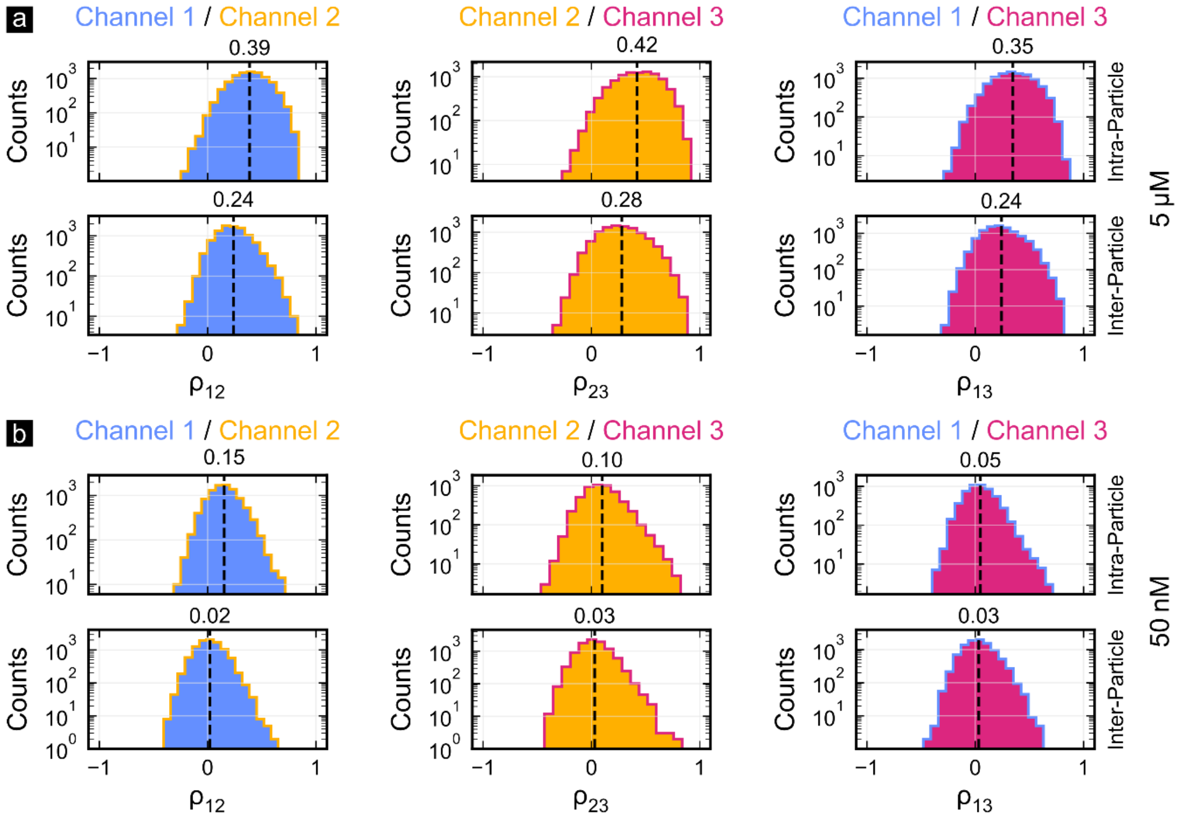

Figure S5. The cross-correlations ρ_{ij} are computed as the correlation between the baseline-corrected time traces of the channels i and j.The intra-particle cross-correlation is calculated for two channels of the same particle (here HCP-AiBs) and the inter-particle cross-correlation for two channels of the two spatially most separated particles of the same array. The time traces are acquired at (a) $c = 5$ μM and (b) $c = 50$ nM of Atto 488, Cy3, and Alexa 647. The dashed vertical lines indicate the mean value of the distribution. The underlying data were recorded at $f = 1.67$ fps ($\delta t = 200$ ms).

three fluorophores are shown together with the transmission windows of the excitation filter T_{ex}. The emission spectra of the fluorophores are shown on top of the transmission windows of the emission filter T_{em}. A suitable dichroic is used to reduce the amount of light lost in the optical path. Using a filter set with four transmission bands allows to sequentially excite and detect the fluorescence of up to four fluorophores with a single camera. Figure S4 (b) shows a schematic of the sequential widefield excitation setup used for the measurements. The sCMOS camera triggers a switch of the excitation channel upon the acquisition of a new frame. This allows to sequentially excite the spectrally separated fluorescence emission of the fluorophores in the HCP-AiB hotspot. Through the widefield excitation and camera-based detection, the signal from over 1000 HCP-AiBs can be detected in parallel. More technical details can be found in the methods section of the manuscript.

5. Fluorescence Cross-Talk Analysis

We calculated the cross-correlation ρ_{ij} between the baseline-corrected fluorescence time traces of two detection channels ($i, j \in \{1,2,3\}$ and $i \neq j$) to determine the fluorescence cross-talk.

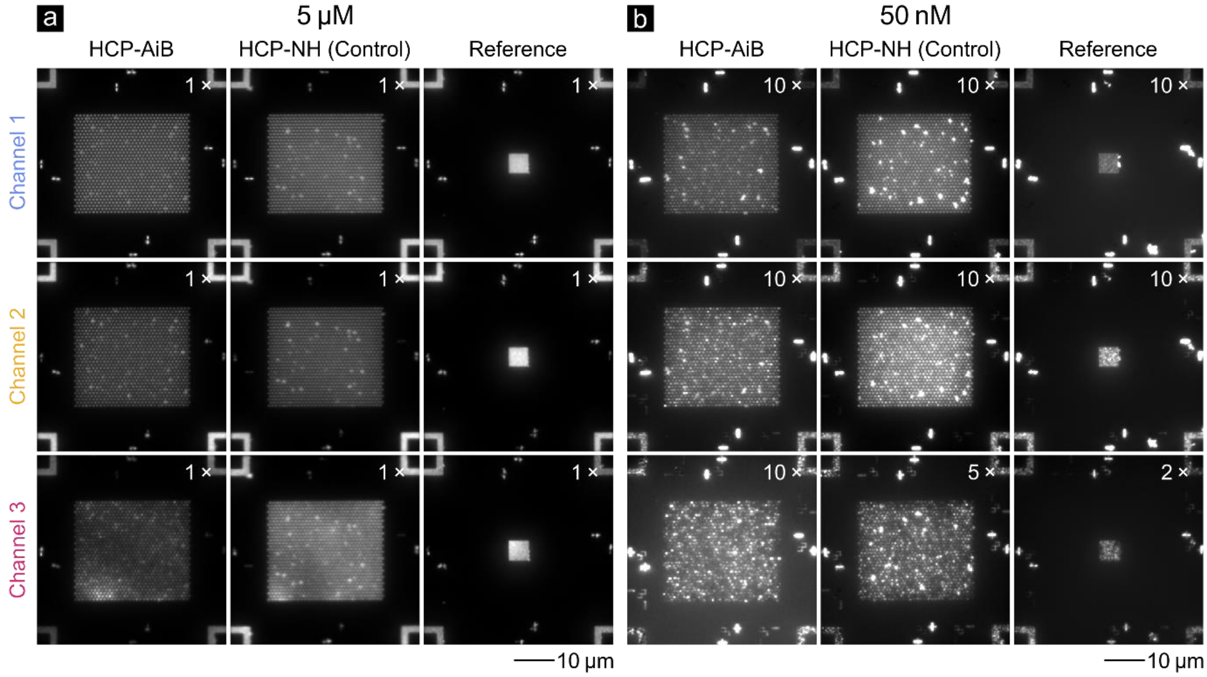

Figure S6. ROIs of HCP-AiB arrays, HCP-NH arrays, and the reference fields are shown for all three excitation channels at (a) $c = 5$ µM and (b) $c = 50$ nM of Atto 488 (channel 1), Cy3 (channel 2), and Alexa 647 (channel 3). The images are clipped and brightness adjusted by the factor shown on the top right for better visibility and were recorded at $f = 1.67$ fps ($\delta t = 200$ ms).

The cross-correlations ρ_{ij} are shown in Figure S5 for (a) $c = 5$ µM and (b) $c = 50$ nM. We distinguish between the intra-particle cross-correlation (same HCP-AiB, distinct detection channels) and inter-particle cross-correlation (distinct HCP-AiBs, distinct detection channels). This allows to better differentiate correlations introduced by fluorescence cross-talk from those originating from other sources such as excitation power fluctuations or stage instabilities. The inter-particle cross-correlation is computed for the two spatially most separated HCP-AiBs within the same array. We distinguish between very weak ($\rho_{ij} = 0.0 - 0.2$), weak ($\rho_{ij} = 0.2 - 0.4$), moderate ($\rho_{ij} = 0.4 - 0.6$), strong ($\rho_{ij} = 0.6 - 0.8$), and very strong ($\rho_{ij} = 0.8 - 1$) correlations.

At $c = 5$ µM we observe weak to moderate correlations for all three detection channels. Particularly for the channels 2 and 3 we find an elevated average cross-correlation of $\langle\rho_{23}\rangle = 0.42$. Considering the spectra shown in Figure S4, we attribute this mostly to the spectral overlap between the Cy3 emission and Alexa 647 absorption spectra. The spectral overlap between the excitation spectra with the absorption spectrum of the other fluorophore (i.e. between channel 2 and Alexa 647 and between channel 3 and Cy3) is rather negligible. However, also the inter-particle cross-correlation shows weak correlations indicating that a

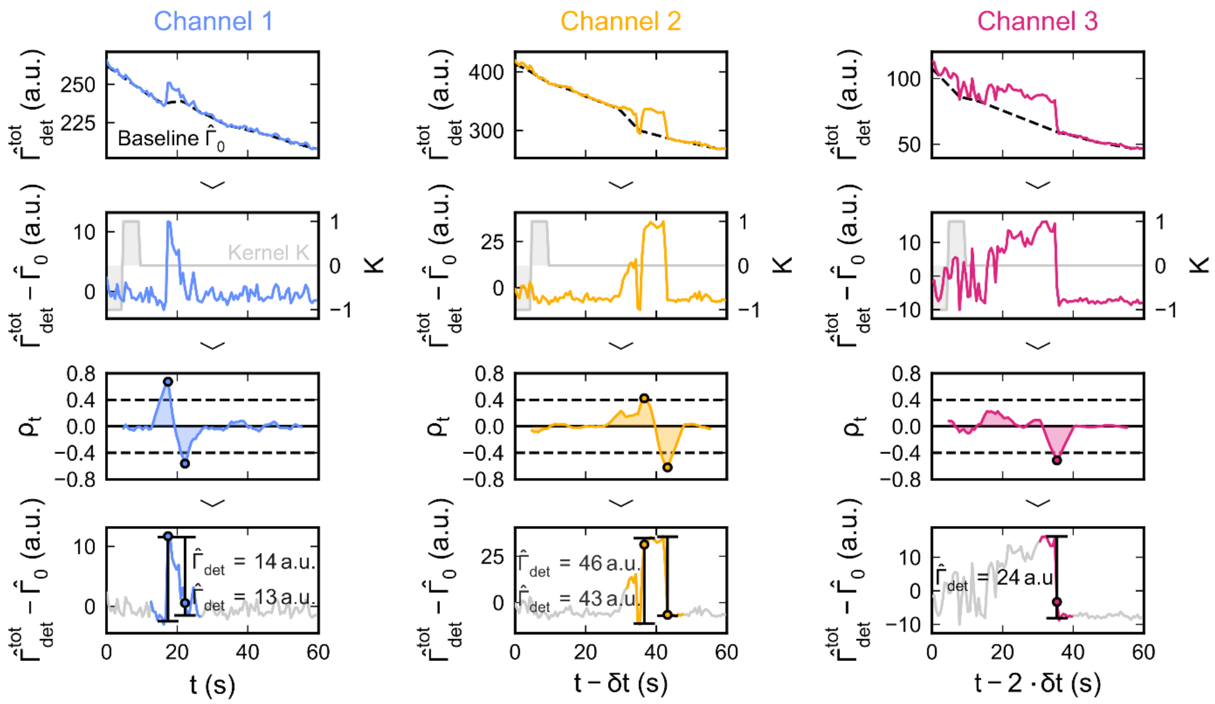

Figure S7. A custom algorithm automatically identifies blinking and bleaching events in the fluorescence time traces extracted from the three excitation channels. For this, (i) the baseline $\hat{\Gamma}_0$ is subtracted from the power normalized time trace $\hat{\Gamma}_{\mathrm{det}}^{\mathrm{tot}}$ and (ii) the baseline corrected time trace is correlated with a kernel K. (iii) Peaks with $|\rho_t| > 0.4$ are detected in the correlation function ρ_t. (iv) The temporal location of these peaks corresponds to the transition point of blinking and bleaching events. Ultimately, the single-molecule fluorescence detection rate $\hat{\Gamma}_{\mathrm{det}}$ is determined from the 10 – 90% signal change within a time window symmetrically positioned around these transition points. These time traces were recorded at $c = 5$ µM and $f = 1.67$ fps ($\delta t = 200$ ms).

relevant portion of the observed correlations does not come from fluorescence cross-talk. We explain the elevated inter-particle cross-correlation mostly by the unspecific exponential decay of the fluorescence backgrounds that remains after applying the baseline correction.

Figure S5 (b) suggests that the fluorescence cross-talk most likely does not influence the single-molecule analyses. This is because the intra-particle cross-correlations are very weak at $c =$ 50 nM, where the fluorescence background is almost absent. This indicates that the correlations are indeed mostly introduced by the unspecific fluorescence background and not by single-molecule emissions from the HCP-AiB hotspots leaking into the other detection channels.

6. Fluorescence Time Trace Analysis

Figure S6 shows the ROIs of the HCP-AiB, HCP-NH, and reference fields for all three detection channels and the two concentrations used in this study. (a) At $c = 5$ µM it is not possible to detect individual molecules in the reference field. (b) At $c = 50$ nM it becomes possible to detect individual fluorophores in the reference fields allowing to infer the fluorescence detection rate enhancement provided by HCP-AiBs and HCP-NHs. Large differences in brightness are observed for the HCP-AiBs and HCP-NHs since at such low concentrations it is not ensured anymore to have a fluorophore in every hotspot.

The fluorescence time traces were extracted from those images and automatically analyzed for blinking and bleaching events using the four steps shown in Figure S7. Initially, (i) the baseline $\hat{\Gamma}_0$ of each power normalized time trace $\hat{\Gamma}_{\mathrm{det}}^{\mathrm{tot}}$ is detected to correct for the bleaching of the fluorescence background using a baseline correction algorithm.[5] Then, (ii) the baseline corrected time trace $\hat{\Gamma}_{\mathrm{det}}^{\mathrm{tot}} - \hat{\Gamma}_0$ is temporally correlated with a kernel K to obtain the temporal correlation function ρ_t. The kernel has a length of 10 s (low-speed imaging) or 3 s (high-speed imaging) and a value of -1 for the first half and of $+1$ for the second half. (iii) Peaks in the correlation function are then identified using a peak finding algorithm. Peaks are required to fulfill $|\rho_t| > 0.4$ and to be separated more than half a kernel length to avoid redundant detections of the same blinking or bleaching event. If more than one peak is detected within that time window, only the biggest one is considered. The temporal location of the detected peaks corresponds to the inflection points of blinking or bleaching events. (iv) An analysis window is placed symmetrically around the detected inflection points in the baseline corrected fluorescence time trace (see colored lines). The length of the analysis window corresponds to the kernel length. Finally, the single-molecule fluorescence detection rates $\hat{\Gamma}_{\mathrm{det}}$ are determined as the $10 - 90\%$ signal change within the analysis window.

7. Considerations for Comparing Fluorescence Enhancement Factors

Caution is necessary when comparing fluorescence enhancement factors across different studies, as various factors can significantly influence the observed enhancement factors.

First, achieving high enhancement factors is generally easier at longer wavelengths (red, near-infrared). This is due to the excellent plasmonic properties of gold, the larger resonance wavelengths yielding a larger dipole moment of the nanoantenna, and the smaller impact of the limited fabrication resolution (e.g., a 20 nm fabrication resolution affects a 200 nm nanoantenna less than a 50 nm one).

Second, enabling multicolor fluorescence detection within the same hotspot requires the plasmonic resonance to span a wide spectral range. This necessitates low Q-factors (resonance wavelength divided by resonance linewidth), which in turn implies lower enhancement factors.

Third, significantly higher enhancement factors can be achieved for fluorophores with low intrinsic quantum yields. This is particularly beneficial for enhancing weak autofluorescence.[6,7] However, it makes the comparison of enhancement factors more challenging when chemical quenchers are used to artificially reduce the quantum efficiency.[8,9]

Fourth, the method of analysis significantly influences the determined enhancement factors. Our approach (extracting single-molecule fluorescence from the blinking/bleaching of statically

embedded fluorophores) provides a direct measure of single-molecule fluorescence. Dynamic methods, which analyze fluorophores in solution, have the advantage of eliminating the influence of fluorophore orientation (as their dipole moment rotates) and increasing the probability of detecting a fluorophore in the hotspot (due to diffusion). However, these methods often require precise knowledge of the size and shape of the plasmonic hotspot, which can be difficult to determine accurately. Many studies assume a Gaussian observation volume, which is an imprecise representation as evidenced by simulations.

Below, we summarize the enhancement factors reported in several studies on fluorescence enhancement with plasmonic nanostructures. We have highlighted in green the studies we consider comparable, as they use fluorophores with relatively high quantum efficiencies (>10%) and similar spectral ranges. The studies highlighted in yellow partially fulfill these criteria (notably, they were conducted in the UV). We consider the studies highlighted in red to be not comparable due to the use of fluorophores with very low quantum efficiencies or chemical quenchers.

Articles	Fluorophores	Excitation Wavelengths	Quantum Efficiencies*	Quenchers	Methods**	Enhance-ments
Our work	Atto 488,<br>Cy3,<br>Alexa 647	~479 nm,<br>~554 nm,<br>~638 nm	80%,<br>31%<br>15%	None,<br>None,<br>None	Static **multicolor** fluorescence blinking/bleaching analysis w. aluminum HCP-AiBs	5,<br>13,<br>14
Bharadwaj (2007)[3]	Alexa 488,<br>Nile Blue	488 nm<br>637 nm	92%,<br>27%	None,<br>None	Static **single-color** count rate analysis w. silver (Alexa 488) and gold (Nile Blue) nanospheres	15,<br>9
Punj (2013)[9]	Alexa 647	633 nm	8%	Chemical	Dynamic **single-color** count rate analysis w. **gold** AiBs	760 – 1,100
Flauraud (2017)[8]	Alexa 647,<br>Crystal Violet	633 nm,<br>633 nm	8%,<br>2%	Chemical,<br>None	Dynamic **single-color** burst analysis w. gold AiBs	5,300,<br>15,000,
Wientjes (2014)[10]	LH2	850 nm	2.5%	None	Static **single-color** count rate analysis w. gold nanorods	23
Kaminska (2018)[11]	Alexa 488	487 nm	92%	None	Static **single-color** fluorescence blinking/bleaching analysis w. silver dimer	30

Barulin (2019)[6]	p-terphenyl, β-galactosidase (tryptophan)	266 nm, 295 nm	93%, <13%	None, None	Dynamic **single-color** count rate analysis w. aluminum ZMWs	4, 5
Roy (2023)[7]	p-terphenyl, Streptavidin, Hemoglobin	266 nm, 295 nm, 295 nm	93%, 3.5%, 0.5%	None, None, None	Dynamic **single-color** count rate analysis w. hybrid aluminum/rhodium AiB-like antennas	~20, 41, 120
Herkert (2023)[4]	Alexa 647	640 nm	15%	None	Static **single-color** fluorescence blinking/bleaching analysis w. hybrid aluminum/gold HCP-AiBs	50

Table S1. Comparison of fluorescence enhancement factors reported across different studies. Background colors indicate the comparability of the conditions under which the enhancement factors were determined: green for comparable conditions, red for non-comparable conditions, and yellow for partially comparable conditions.
* Quantum efficiencies are looked up online if not found in the articles.
** Experiments are only considered multicolor when multiple fluorophores are detected simultaneously.

References

1 Anger P, Bharadwaj P, Novotny L. Enhancement and quenching of single-molecule fluorescence. *Phys Rev Lett* 2006; **96**: 3–6.

2 Abd El-Fattah ZM, Mkhitaryan V, Brede J, Fernández L, Li C, Guo Q *et al.* Plasmonics in Atomically Thin Crystalline Silver Films. *ACS Nano* 2019; **13**: 7771–7779.

3 Bharadwaj P, Novotny L. Spectral dependence of single molecule fluorescence enhancement. *Opt Express* 2007; **15**: 14266.

4 Herkert EK, Bermeo Alvaro DR, Recchia M, Langbein W, Borri P, Garcia-Parajo MF. Hybrid Plasmonic Nanostructures for Enhanced Single-Molecule Detection Sensitivity. *ACS Nano* 2023; **17**: 8453–8464.

5 Zhang ZM, Chen S, Liang YZ. Baseline correction using adaptive iteratively reweighted penalized least squares. *Analyst* 2010; **135**: 1138–1146.

6 Barulin A, Claude JB, Patra S, Bonod N, Wenger J. Deep Ultraviolet Plasmonic Enhancement of Single Protein Autofluorescence in Zero-Mode Waveguides. *Nano Lett* 2019; **19**: 7434–7442.

7 Roy P, Zhu S, Claude JB, Liu J, Wenger J. Ultraviolet Resonant Nanogap Antennas with Rhodium Nanocube Dimers for Enhancing Protein Intrinsic Autofluorescence. *ACS Nano* 2023; **17**: 22418–22429.

8 Flauraud V, Regmi R, Winkler PM, Alexander DTL, Rigneault H, Van Hulst NF *et al.* In-Plane Plasmonic Antenna Arrays with Surface Nanogaps for Giant Fluorescence Enhancement. *Nano Lett* 2017; **17**: 1703–1710.

9 Punj D, Mivelle M, Moparthi SB, Van Zanten TS, Rigneault H, Van Hulst NF *et al.* A plasmonic 'antenna-in-box' platform for enhanced single-molecule analysis at micromolar concentrations. *Nat Nanotechnol* 2013; **8**: 512–516.

10 Wientjes E, Renger J, Curto AG, Cogdell R, Van Hulst NF. Nanoantenna enhanced emission of light-harvesting complex 2: The role of resonance, polarization, and radiative and non-radiative rates. *Phys Chem Chem Phys* 2014; **16**: 24739–24746.

11 Kaminska I, Vietz C, Cuartero-González Á, Tinnefeld P, Fernández-Domínguez AI, Acuna GP. Strong plasmonic enhancement of single molecule photostability in silver dimer optical antennas. *Nanophotonics* 2018; **7**: 643–649.
